# Impact of Stress on Brain Morphology: Insights into Structural Biomarkers of Stress-related Disorders

**DOI:** 10.2174/1570159X21666230703091435

**Published:** 2023-07-03

**Authors:** Narcís Cardoner, Raül Andero, Marta Cano, Ignacio Marin-Blasco, Daniel Porta-Casteràs, Maria Serra-Blasco, Esther Via, Muriel Vicent-Gil, Maria J. Portella

**Affiliations:** 1 Institut d'Investigació Biomèdica Sant Pau (IIB SANT PAU), Hospital de la Santa Creu i Sant Pau, Barcelona, Spain;; 2 Department of Psychiatry and Forensic Medicine, School of Medicine Bellaterra, Universitat Autònoma de Barcelona, Barcelona, Spain;; 3 Centro de Investigación Biomédica En Red en Salud Mental (CIBERSAM), Instituto de Salud Carlos III, Madrid, Spain;; 4 Unitat de Neurociència Traslacional, Parc Taulí Hospital Universitari, Institut d'Investigació i Innovació Parc Taulí (I3PT), Institut de Neurociències, Universitat Autònoma de Barcelona, Cerdanyola del Vallès, Spain;; 5 Institut de Neurociències, Universitat Autònoma de Barcelona, Cerdanyola del Vallès, Barcelona, Spain;; 6 Departament de Psicobiologia i de Metodologia de les Ciències de la Salut, Universitat Autònoma de Barcelona, Cerdanyola del Vallès, Barcelona, Spain;; 7 ICREA, Barcelona, Spain;; 8 Programa eHealth ICOnnecta't, Institut Català d'Oncologia, Barcelona, Spain;; 9 Child and Adolescent Psychiatry and Psychology Department, Hospital Sant Joan de Déu, Barcelona, Spain;; 10 Child and Adolescent Mental Health Research Group, Institut de Recerca Sant Joan de Déu, Barcelona, Spain

**Keywords:** Brain morphology, stress response systems, basolateral amygdala, PTSD, HPA

## Abstract

Exposure to acute and chronic stress has a broad range of structural effects on the brain. The brain areas commonly targeted in the stress response models include the hippocampus, the amygdala, and the prefrontal cortex. Studies in patients suffering from the so-called stress-related disorders -embracing post-traumatic stress, major depressive and anxiety disorders- have fairly replicated animal models of stress response -particularly the neuroendocrine and the inflammatory models- by finding alterations in different brain areas, even in the early neurodevelopment. Therefore, this narrative review aims to provide an overview of structural neuroimaging findings and to discuss how these studies have contributed to our knowledge of variability in response to stress and the ulterior development of stress-related disorders. There are a gross number of studies available but neuroimaging research of stress-related disorders as a single category is still in its infancy. Although the available studies point at particular brain circuitries involved in stress and emotion regulation, the pathophysiology of these abnormalities -involving genetics, epigenetics and molecular pathways-, their relation to intra-individual stress responses -including personality characteristics, self-perception of stress conditions…-, and their potential involvement as biomarkers in diagnosis, treatment prescription and prognosis are discussed.

## 1 INTRODUCTION

Stress-related disorders embrace a group of clinically distinct but strongly interlinked disorders ranging from post-traumatic stress and anxiety to major depressive disorders [1, 2]. With a high estimated global and lifetime prevalence [1, 2], it significantly contributes to the world disease burden and disability [3, 4]. This impact has even been magnified in the context of the global pandemic, a highly stressful-laden scenario, where increased prevalence has been notably detected [5]. While stress is a normal human reaction, research on the biology of stress has extensively shown that body and brain health can be derailed by excessive or prolonged activation of stress response systems. The so-called ‘toxic stress’ [6] has therefore been considered to damage the architecture of the developing brain [7] and to alter structural and functional areas of the mature central nervous system.

Along with Post-traumatic Stress Disorder (PTSD), Major Depressive Disorder (MDD) and Anxiety Disorders are commonly referred to as ‘stress-related disorders [8, 9]. Beyond having acute and chronic stress as a precipitating factor, they share many features in their clinical expression and display significant comorbidities among them. Indeed, a common genetic etiology has been suggested to account for the comorbidity among stress-related disorders as family and twin studies have described substantial heritability (above 25%) and shared genetic liability [10-12]. To further support such commonalities, there exist shared pharmacological response patterns, *i.e*., both SSRIs and SNRIs are first-line options for the treatment of PTSD, major depression, or anxiety disorders. Although these disorders also present specific dimensions that might not be shared among them, which confer different subtypes within each diagnostic category, especially MDD, possibly casting doubt on such common neurobiological underpinnings [13-15], multidimensional models derived from comprehensive descriptions of micro to macro factors may represent an opportunity [16].

Herein, this narrative review aims at determining whether structural brain changes are truly common across stress-related disorders, by describing the most accepted neurobiological mechanisms of animal models that may be underneath the most consistent structural neuroimaging findings in the developing brain, PTSD, MDD, and anxiety disorders. Based on a previous meta-analysis published on structural brain correlates in major depression, anxiety disorders, and post-traumatic stress disorder [17] a non-comprehensive selection of studies that complemented the findings of this meta-analysis was carried out in order to discuss to what extent the effects of stress in animal models converge into identifiable changes in brain structure, what is the potential mechanism involved, and whether these changes are akin to those observed in humans. A literature search of structural neuroimaging studies including subjects with normal developing brains, PTSD, MDD, and anxiety disorders was conducted in PubMed, Web of Knowledge, ScienceDirect, and Scopus databases. The search keywords were: morphometry OR structural OR VBM OR voxel-wise AND depression OR anxiety disorder OR panic disorder OR phobia OR agoraphobia OR stress disorder OR posttraumatic stress disorder. For animal models of stress-related disorders, a separate search was performed provided that the previous search scarcely detected any study. The keywords were: Animal models AND stress-related disorders OR depression OR anxiety OR post-traumatic stress disorder AND HPA axis OR glucocorticoids OR chronic stress OR acute stress OR proinflammatory cytokines OR brain structural changes.

## 2 ANIMAL MODELS OF STRESS-RELATED DISORDERS

Some of the critical factors in animal models that could define the threshold for the onset of phenotypes compatible with the symptoms of stress-related disorders are the stress type, severity, duration, frequency, variability, and predictability of stress, with special attention to the individual differences in stress perception [18-21]. In general, it is considered that acute stress elicits PTSD-like animal models, chronic exposure to stress evokes MDD-like models [18, 22] whereas approach-avoidance situations and conditioned fear responses, anxiety-like animal models [23].

PTSD-like models are based on physical or psychological stressors applied individually or in combination. Some of the criteria for the validity of PTSD translational models proposed by Yehuda & Antelman [20] are PTSD-like biological and behavioural outcomes, intensity-dependent responses, and persistence of the PTSD-like phenotype over time. Additionally, severe and predominantly emotional stressors seem to be more adequate since stressors with a prominent physical component, such as foot or tail shocks, can cause injuries that could affect motivation and locomotion altering behavioural measures [24]. For instance, immobilization (IMO) is a severe predominantly emotional stressor in rodents as measured by the physiological responses it elicits [25, 26] and its acute application has been shown to induce long-term consequences in behavior and memory compatible with PTSD symptoms [26-28]. In addition, PTSD-like effects of IMO have been associated with changes in synaptic plasticity in the hippocampus [29] and medial prefrontal cortex (mPFC) [30].

The use of animal models has been crucial for the understanding of the neuroanatomy, neurophysiology, and the molecular processes underlying MDD [31-34]. However, the validity of animal models of MDD has been classically based on the responses to antidepressant treatments [31] thus leading to a poor understanding of other relevant criteria. Currently, animal models of MDD are mainly considered when showing responses to antidepressants such as selective serotonin or noradrenaline reuptake inhibitors, or tricyclic antidepressants. Animal models of MDD are based on chronic exposure to stressors reproducing adverse life events during the developmental period or adulthood. Among them, chronic exposure to social defeat causes behavioural, neurobiological, hormonal, and neurochemical changes compatible with MDD symptoms [35-41]. The application of the unpredictable chronic mild stress paradigm, consisting of the aleatory exposure to different mild stressors during several weeks of, also mirrors changes observed in MDD such as an increased hypothalamic-pituitary-adrenal (HPA) axis sensitivity and a decrease in responses to pleasant stimuli [42]. However, the use of this model is controversial due to its reproducibility [43].

Different behavioural tests such as the elevated plus maze or the open field are frequently referred to as models of anxiety disorders in the literature. However, the formers are used to measure the anxiety response, while an animal model should elicit a phenotype compatible with the pathology [44]. Anxiety disorders are characterized by fear reactions in situations where such fear is not justified. In this sense, fear conditioning seems to be a good model for anxiety disorders since in this paradigm, an aversive unconditioned stimulus, such as a shock, conditions a potent and permanent fear reaction to a harmless stimulus [45].

### 2.1 Molecular Mechanisms Underlying Brain Structural Changes

#### 2.1.1 Neuroendocrine Model

Alterations in brain structure induced by exposure to physical or psychological stressors seem to be primarily due to an overactivation of the HPA axis and the consequent increase in the release of glucocorticoids [46]. A pivotal finding is that chronic exposure to glucocorticoids induced by stress can lead to long-term effects on brain structure. Several studies have shown that the treatment with exogenous glucocorticoids in non-stressed animals can trigger changes in brain structure in a region-specific manner. For example, AdKO transgenic mice, an animal model of Cushing’s syndrome whose main symptom is long-term hypercorticosteronemia, showed reduced relative volumes in multiple brain regions such as the corpus callosum and several cortical areas and increased volume in the medial amygdala, bed nucleus of the stria terminalis, and hypothalamus [47]. These changes are explained by aberrant myelination and white matter damage [47]. Chronic administration of corticosterone results in dendritic reorganization or atrophy in pyramidal neurons of the mPFC [48, 49]. Also, chronic glucocorticoid treatment produces hippocampal dendritic shrinkage in rats and primates [50, 51]. Similarly, a single acute dose of corticosterone is sufficient to induce dendritic hypertrophy in the basolateral amygdala (BLA) in a magnitude like that caused by chronic treatment [52]. The inhibition of the synthesis of glucocorticoids in animals submitted to repeated restraint stress can prevent hippocampal dendrite atrophy induced by this stressor [53].

Most of the studies linking stress-induced changes in brain structure are focused on the hippocampus due to its particular sensitivity to chronic stress and glucocorticoids, its critical role in learning and memory, and also its participation in the negative feedback regulation of the HPA axis [54-57]. Other regions such as the mPFC and the amygdala have received attention due to their relevance in the stress response and their role in processing, acquisition, and extinction of fear memories [58]. For instance, chronic exposure to different stressors such as restraint or social defeat can cause shortening and debranching in apical dendrites of hippocampal CA3 pyramidal neurons [53, 59-64]. The deleterious effect of glucocorticoids in the hippocampus in terms of dendritic atrophy has been explained by different hypotheses [65]. In one hand the Glucocorticoid Cascade Hypothesis formulated by Sapolsky and colleagues [66] suggested that the excess of glucocorticoids and the downregulation of glucocorticoid receptors induced by chronic stress activate a feedback reaction leading to dendritic retraction, degeneration, and a consequent disease. On the other hand, the Glucocorticoid Vulnerability Hypothesis [67] suggests that the dendritic retraction-induced prolonged or repeated exposure to glucocorticoids induced by a chronic stress history, makes the hippocampus vulnerable to subsequent neurotoxic or metabolic challenges. Both hypotheses are complementary and provide a plausible explanation for the genesis and progression of brain structural changes induced by stress exposure.

#### 2.1.2 Glutamatergic Model

The available data set seems to confirm that glucocorticoids interact synergistically with glutamatergic neurotransmission, neurotrophin-related signaling, and proinflammatory cytokine signaling to induce structural changes in the brain after stress exposure [68-71]. Changes in glucocorticoid levels induced by circadian rhythms and stress alter the basal release of glutamate in several areas, such as the hippocampus, amygdala, and prefrontal cortex (PFC) [72, 73]. This effect has been observed after exposure to different stressors or administration of glucocorticoids and it can be reverted by adrenalectomy [72, 74-77]. Hippocampal CA3 neuron atrophy induced by chronic restraint stress can be reverted by the administration of an antagonist of N-methyl-D-aspartate (NMDA) receptors [53]. Moreover, NMDA receptor blockade prevents chronic stress-induced dendritic shrinkage of mPFC neurons [78]. Neurotrophins such as the brain-derived neurotrophic factor (BDNF), one of the most abundant neurotrophic factors in the brain, participate in these structural changes through their union with neurotrophin receptors. The binding of BDNF to the tyrosine receptor kinase B (TrkB) promotes its interaction with NMDA receptors in the hippocampus and PFC leading to changes in the expression of immediate early genes such as the activity-regulated cytoskeleton-associated protein (Arc) [79-82]. These TrkB-mediated gene expression changes can influence processes such as neurogenesis, synapse formation, and plasticity [83-89]. Importantly, several members of TrkB downstream signaling cascades are altered in MDD and other mood disorders patients [90-92]. Similar changes in terms of gene expression are also observed in the hippocampus and the PFC of animals submitted to stress [93-96].

#### 2.1.3 Inflammatory Model

Proinflammatory cytokines such as IL-6, IL-1β, and TNF-α are induced by psychological stress and are implicated in PTSD, anxiety disorders, and MDD [16, 97-101]. Brain areas repeatedly associated with these stress-related disorders such as the hippocampus, amygdala, and anterior cingulate cortex (ACC) have been reported to be particularly influenced by pro-inflammatory cytokines [102]. The increase in pro-inflammatory cytokines decreases neurogenesis in the hippocampus by down-regulating the BDNF pathway [103-105]. Glutamate can also be increased by pro-inflammatory cytokine levels leading to excitotoxicity and reduced neurogenesis through the activation of NMDA receptors [106, 107]. Pro-inflammatory cytokines can also induce the release of reactive oxygen species by astrocytes and microglia promoting neuronal oxidative damage [108, 109]. Other inflammatory markers such as C-reactive protein (CRP), whose levels are increased after IL-6 secretion [110], have also been reported to be elevated in patients with MDD [111, 112]. CRP has been reported to induce reactive gliosis that can exert detrimental effects on astrocytes and microglia [113]. CRP also mediates the decrease in neurogenesis in the PFC and hippocampus of rats exposed to chronic unpredictable stress [114]. Pro-inflammatory cytokines also promote an alternative tryptophan metabolism pathway, different from that which metabolizes tryptophan to serotonin and melatonin, named the kynurenine pathway. Dysregulation of the kynurenine pathway may deplete serotonin in the CNS, which is closely related to the pathophysiology of MDD [115]. Kynurenine pathway metabolites such as 3-hydroxy-kynurenine, 3-hydroxy-anthralinic acid, and quinolinic acid are neurotoxic and can trigger neuronal apoptosis through oxidative stress or altering glutamatergic neurotransmission [116].

### 2.2 Neuroimaging Findings in Stress-related Animal Models

Evidence derived from animal models on stress-related conditions have provided the molecular mechanisms which in turn would shape the brain structure alterations described across several neuroimaging studies of the so-called stress-related psychiatric disorders. However, probably due to technical limitations, few studies on animal models have addressed longitudinal stress-induced changes in structural integrity. Post-mortem high-resolution structural MRI and diffusion kurtosis imaging in rats exposed to chronic immobilization stress found dendritic morphology changes in the hippocampus, amygdala, and mPFC [117]. Also, *ex-vivo* MRI in mice exposed to chronic social defeat stress showed changes in areas modulators of the HPA axis such as the hippocampus and the bed nucleus of the stria terminalis, and areas related to the development of depressive-like behaviour and fear management such as the raphe nuclei and periaqueductal grey matter [118, 119]. The first longitudinal MRI study in the literature assessing the impact of chronic unpredictable stress in rats revealed structural atrophy in key regions such as the prelimbic, cingulate, insular and retrosplenial, somatosensory, motor, auditory and perirhinal/entorhinal cortices, the hippocampus, the dorsomedial striatum, nucleus accumbens, the septum, the bed nucleus of the stria terminalis, the thalamus, and several brain stem nuclei [120]. Subsequent longitudinal studies using Diffusion Tensor Imaging (DTI) showed no evidence of stress-induced brain volumetric alterations. However, they point to microstructural alterations (fractional anisotropy, mean diffusivity, and radial diffusivity values) in the hippocampus of mice exposed to chronic social defeat [121] and the corpus callosum, external capsule, anterior commissure, and the amygdala of rats exposed to chronic immobilization [122].

## 3 STRESS AND THE NEURODEVELOPMENTAL BRAIN

In the search for a better comprehension of early brain changes leading to stress-related psychiatric disorders, it has become imperative to study normal brain development, how stress during this period affects brain architecture, as well as how much risk (or protection) it contributes to the development of a stress-related disorder. The relevance of such studies is evidenced when considering that half of the mental health disorders emerge by the age of 14 years old [8] and that early life stress is suggested to explain between 20-30% of the variance in mental health disorders [123].

During human brain development, stress is necessary for building and shaping the repertoire of responses to environmental changes, with multiple complex brain processes subserving such behaviors. At the neuronal level, underlying mechanisms involve neurogenesis, neuronal migration, synaptogenesis, synaptic remodeling, synaptic pruning, myelination, and apoptosis, which follow time-overlapping sequences that are specific for each region and that are at a maximum growth rate during the 2^nd^-3^rd^ pregnancy trimesters and the first two years of life [124-126]. They constitute the basis of neurotransmitters’ communication systems formation, which goes through immature stages during early adolescence to reach the adult brain, being relevant for this period of the development in the balance between excitatory and inhibitory neurotransmission [124-126]. All these processes serve to build brain regions and the connectivity paths between them, finally to develop networks underlying specific functions [126]. Excessive or prolonged activation of the stress response during development might impair brain architecture and function, with possible consequences into adulthood. The effects of stress on neurodevelopment are dependent on the characteristics of stress but also the time they are produced: during sensitive periods the response to experiences is heightened, while in critical periods the changes that occurred (or the lack of them) might be irreversible [127]. Anyhow, periods of increased change for each area, detailed below, are considered more vulnerable to insults [127].

Brain circuit development follows a established and organized sequence, with sensorial and thereafter motor regions maturing earlier than regions involved in higher cognitive processes [126, 128]. This translates into a general posterior-to-anterior sequence of maturation that finishes in parietal regions for the posterior half of the brain and in the dorsolateral PFC (DLPFC) for the anterior part [128]. The DLPFC, the posterior part of the superior temporal gyrus (STG), and the posterior parietal cortex are the last areas to mature by early adulthood [126, 128]. For specific networks, subcortical structures are also observed to mature earlier than cortical ones [126]. Gray matter (GM) shows a high growth rate during the first two postnatal years being maximum at the cerebellum, subcortical areas, and the cerebral cortex [126, 129]; this growth rate is sustained during childhood and decreases after 10 years old, with some sex-related differences [124, 126, 130]. White matter follows a marked increase between early childhood and adolescence, reaching its peak around 45 years old [124] associated with myelination processes, axon caliber, or both [128]. A process described as vulnerable to psychopathology is the loss of GM in DLPFC that occurs in late adolescence -associated with synaptic plasticity and pruning- which concurs with white matter growth in the PFC, ACC, and temporal poles [124, 126, 128]. As a consequence of region-specific development, there is an asymmetric development in adolescence between prefrontal control systems and regions of limbic and reward systems -the latter maturing earlier than the former-, which is associated with behavioral characteristics of this period, such as increased risk-taking behaviors [131].

Stress might impact brain morphology or its normal development trajectory [132]. Given the relevance of the HPA axis and corticoids in stress regulation, regions commonly studied are the ones with a role in HPA regulation, *i.e*.: the hippocampus, the amygdala, and the PFC [132], which among other functions are critical for processes of fear extinction and emotion regulation [133]. Balanced regulation of glucocorticoids as well as other molecules involved in inflammation -*i.e*., cytokines- is required for normal neurodevelopment of axonal terminal maturation, axonal growth, and remodulation, among others. By contrast, external insults such as chronic stress exposure alter normal functioning by increasing glucocorticoids levels and downregulating glucocorticoid receptors, which impact directly the shape and function of the key structures, especially the hippocampus, as discussed in-depth in the preceding section [132, 134, 135]. Several studies have evaluated the impact of early life stress on brain maturation during pregnancy or after traumatic events during childhood. During prenatal life, events such as infection, drug use, environmental exposure, hypoxia, ischemia, hypoperfusion and maternal-related health condition, maternal stress, or maternal malnutrition are thought to produce a widespread reorganization of large-scale connections [136]. Some specific alterations are also found, although with contradictory findings [136]. For example, increased maternal exposure to stressful events, maternal stress, and maternal anxiety traits are associated with greater right amygdala in newborns and greater fronto-limbic structural connectivity at 12 months [135], and lower hippocampal volume or slower hippocampal growth in the first postnatal months on the offspring [129, 135, 136]. Air pollution during pregnancy shows similar results in the offspring: smaller hippocampus but increased amygdala and cerebellum volumes [137]. However, these effects were attributable to decreased global GM volume associated with a low socioeconomic environment [138] in another study. A large number of studies o childhood trauma give evidence of its consequences over the development of regions and networks involved in threat processing and emotion regulatory systems, contributing risk for the emergence of stress-related psychiatric disorders [139]. Most consistently reported brain regions affected by childhood maltreatment are the PFC in its ventromedial parts (vmPFC), the DLPFC, the ACC, the hippocampus, the amygdala, the corpus callosum and the cerebellum [131].

### 3.1 Hippocampus

The hippocampus has received great interest, but results are not exempted from confounders. Reduced hippocampal volume has been observed in adults with a history of childhood trauma [140], suggesting a larger impact when the traumatic event occurred prior to age 14 [139, 141]. In prospective studies, some have found decreased hippocampal volume in maltreated children [142, 143] or no differences in others [144], while a meta-analysis showed a small effect of changes that vanished when controlling by gender [145]. The type of maltreatment is suggested to contribute to differences in findings, with some evidence that threat-related maltreatment (as opposed to deprivation) is associated with neurodevelopmentally decreased hippocampal volume [144, 146]. In other studies, maltreatment was associated with larger left [147] or right [148] hippocampal volumes that followed a flatter growth through mid-adolescence [147-149], which could explain lower hippocampal volumes emerging sometime after maltreatment. An important confounding factor is the presence of psychopathology [145]: changes in growth development (flat growth or delayed maturation) were mediated by the presence of comorbid psychiatric disorders (mostly internalizing -mood or anxiety-) [147] or was observed in a group of abused females that developed anxiety, PTSD or a depressive disorder [146]. Other authors indicate that low hippocampal volume is more associated with the emergence of PTSD rather than trauma itself and suggested a continuum between trauma and PTSD [131, 150]. Low hippocampal volume is additionally observed in non-maltreated children that developed depression [151]; different mechanisms, including the combination of childhood maltreatment and genetic vulnerability, might contribute to low hippocampal volumes that are also present in different stress-related disorders [133, 152].

### 3.2 Amygdala

The amygdala is one of the structures following a slower development over the lifespan [140]. Chronic stress increases dendritic arborization and volume [132]; increased volume is shown in studies of children exposed to maternal deprivation (*i.e*. previous institutionalization, maternal depression) or exposure to adversities or maltreatment [142, 147, 148]. Alike to the hippocampus, findings have been inconsistent, with other studies showing decreases in volume [142] or even no changes in others [145]; the specific finding of decreased amygdalar volume, it is considered to be associated with the presence of PTSD, not to the sole effects of maltreatment [131]. The increase in volume is suggested as an earlier structural maturation to compensate for the absence of an available caregiver [142]; indeed, different studies support the idea that adversity during childhood might produce an accelerated maturation of brain circuits [142-144, 153], which in turn is consistent with clinical observations [154] and studies in animal models [142] as described above. In this regard, one replicated finding is an early maturation in the amygdala-prefrontal connectivity: the expected shift between positive to negative coupling between these structures matures earlier in exposed than non-exposed children [142, 153], and is suggested to be a protective factor for psychopathology [155]. An acceleration in brain maturation, however, might be dependent on other factors such as sex: in non-maltreated children at risk for depression, the emergence of depression is associated with an exaggerated amygdalar growth in females but an attenuated growth in males [151]. Other confounders include the type and timing of stressful exposure, which may produce either a delayed or accelerated maturation of circuits [142, 146].

### 3.3 Prefrontal Cortex

The PFC is a region with high susceptibility to impairment due to its time-prolonged long-lasting development [126]. Volume reductions are observed in the vmPFC, the DLPFC, and the ACC [133, 140, 143, 144, 156], which might coexist with an increased functional maturation [142]. Volume reductions in the vmPFC have also been found in PTSD even when compared to maltreated children without PTSD and controls [148].

### 3.4 Other Regions

Other regions have early evidence of its impairment by stress but are potential targets for future studies. For example, decreased cerebellar volumes in children and adolescents with a history of maltreatment and PTSD is a consistent finding, although it is unclear the effect of trauma alone on the cerebellum [131, 143, 157]. Emergent evidence points to the basal ganglia and insular cortex as areas impaired by early live stressful events [156, 158]. GM reductions of visual areas are reported when after exposure to insufficient care, a reactive attachment disorder emerges [159, 160]. Other mechanisms associated with the emergence of stress-related disorders in youth, and their consequences at the brain level are yet poorly understood. For example, in generalized anxiety disorder in youths, findings suggest GM decreases in the ventrolateral PFC, but the volume increases in other regions, including the amygdala, ACC, precentral cortex, precuneus, putamen, and the STG, while hippocampus volume would be preserved [161].

Although experimental and clinical studies have shown that early life stress may have pervasive and persistent effects on frontal cortical-subcortical circuits, other factors have to be involved in the pathophysiology mechanism to give rise to different clinical manifestations and brain alterations across stress-related disorders. The so-called environmental factors, including early and late life stressors, activate the adaptive physiological mechanism in living organisms by means of the stress system, which can provide balanced or altered responses depending on how (genetically) vulnerable the organism is. Several studies have shown altered activity (both hyper- and hypoactivation) of the stress system in individuals with PTSD, anxiety disorders, and depression. The existing literature has connected early life stressors or traumatic experiences in the past to the development of such psychiatric conditions. However, to date, the link between both phenomena seems to be a matter of speculation as the underlying pathophysiological mechanisms are not fully understood as yet. Or maybe not that speculative, if one takes a deep sight at the brain structural alterations across the psychiatric conditions encompassed within stress-related disorders, as it will be argued in the next sections.

## 4 POST-TRAUMATIC STRESS DISORDER

PTSD is a debilitating psychiatric disorder characterized by intrusive thoughts, avoidance, and hypervigilance following traumatic events. Due to its undeniable relationship to traumatic stress events and with stress-based fear conditioning responses, PTSD represents a landmark disorder for exploring the brain structural impact of stress. This distinctive feature allows us to disentangle rather more precisely whether brain structural changes constitute patterns related to acquired disease or, on the contrary, to stress, since we can compare PTSD patients with healthy individuals who have been exposed to a traumatic event (trauma-exposed) and with those who have not been exposed to a traumatic event (non-trauma-exposed).

### 4.1 Hippocampus

Advances in MRI offered insight into the role the hippocampus may have in PTSD as this brain structure is responsible for the ability to store and retrieve memories and plays a role in a person's ability to overcome fear responses. Both glucocorticoid and inflammatory hypotheses would explain the affectation of the hippocampus in PTSD: The increase in cortisol signals the immune system, which releases inflammatory cytokines, which in turn can activate microglia. These in turn switch from the production of serotonin to a higher production of glutamate that can lead to hippocampus cell damage or death. The majority of early volumetric magnetic resonance imaging (MRI) studies conducted a region-of-interest analysis, focusing on the distinct brain regions of the “fear circuit” described in previous preclinical studies. For instance, Woon and collaborators [162] performed a meta-analysis on 39 hippocampal volumetric studies, and as expected, they identified significant hippocampal volume reductions in patients with PTSD. In the end, the cumulative evidence derived from these preliminary studies conclude with the classical neurobiological PTSD models involving the hippocampus in the pathophysiology of PTSD [163]. It should be noted that this region has been reported independently across other stress-related disorders [9]. Although these hypothesis-driven methods are widely used, they are explicitly characterized by neglecting other brain regions capable of offering the specificity needed for prediction, diagnoses, and personalized PTSD treatments. This fact concerning the hippocampus extends to other groups of regions included in the fear circuitry such as the amygdala and ACC.

### 4.2 Amygdala

Although one of the most known neurocircuitry models of PTSD posits that the amygdala would be hyperresponsive which could not be inhibited by an altered hippocampus, there is no clear evidence for abnormal volumes in PTSD. Amygdala hyperactivation has been reported during several traumatic and non-traumatic situations in PTSD and has been positively correlated with symptom severity [164]. Further studies should investigate the lack of relation between activation and volume of the amygdala.

### 4.3 Prefrontal Cortex

Several structural MRI studies reported decreased volumes of PFC in PTSD, and in particular, few of them explored the mPFC [164]. The ACC volumes appeared to be smaller in patients with PTSD compared to trauma-exposed healthy subjects, and inversely correlated with symptom severity. Indeed, a recent whole-brain voxel-brain morphometry metanalysis investigating brain volumetric correlates in major depression, anxiety disorders, and PTSD has revealed that patients with PTSD showed smaller gray matter volume (GMV) in the left lingual gyrus and the bilateral superior frontal gyrus in comparison to healthy participants [17]. These findings pointed out that the structures critically related to primary stress responses (that is, hippocampus, amygdala, and ACC) may not be the only regions involved in the inherent complexity of PTSD.

The relevance of a design involving groups of healthy participants with and without exposure to traumatic stress is highlighted by two different studies [165, 166] that showed GMV reductions in the superior frontal gyrus as well as in the lingual gyrus when comparing patients with PTSD and non-traumatized healthy participants. In contrast, other publications [167, 168] that assessed patients with PTSD and traumatized controls found a GMV reduction in the superior frontal gyrus but not in the lingual gyrus. Considering that GMV reductions observed in patients with PTSD in comparison to non-traumatized healthy participants but not to traumatized controls may be stress-related, these findings emphasize the superior frontal gyrus as a potentially relevant disease-specific biomarker of PTSD.

### 4.4 Other Regions

The study by Nardo and colleagues [167] also found GM volume reductions in the parahippocampal gyrus, similar to the work with patients with PTSD and traumatized controls by Zhang and colleagues [169]. Although in the meta-analysis by Serra-Blasco and collaborators [17] parahippocampal reductions were only observed when applying a liberal threshold, a more recent whole-brain meta-analysis [170] assessing 122 patients with PTSD and 128 traumatized controls revealed GMV reductions in the parahippocampal gyrus as one of the main results.

Numerous adverse sequelae have been linked to childhood maltreatment, and the literature has unequivocally established that experiences of childhood trauma are associated with significantly increased rates of PTSD across the lifespan. Specifically, some studies have demonstrated structural alterations in both children and adults whose PTSD is secondary to childhood maltreatment [171, 172] such as volumetric reductions in cerebellum, corpus callosum, total cerebral and intracranial volume when compared both to healthy controls and to maltreated individuals without PTSD. However, studies on these other regions are extremely limited to date, and further research is needed to substantiate the theoretical models involving the cerebellum and the corpus callosum.

## 5 MAJOR DEPRESSIVE DISORDERS

Considered to be the most common psychiatric illness, MDD is recognized as a heterogeneous and highly complex disorder, with a recurrent and episodic course often leading to pronounced functional interference during the acute episode and, in a significant number of cases, sustained impairment. Altered responses to chronic stressful events, such as sustained childhood maltreatment and long-term stressful life events, have been considered central to the mechanisms involved in the aetiology of MDD. Based on findings from several animal models, neuroendocrine, neuroinflammatory, and neurotransmitter system responses are the consequence of this defective response to chronic stress, which may ultimately lead to changes in brain structure.

Although there is considerable heterogeneity in terms of the brain areas involved, 25 years of research in this field has laid the groundwork for the relationship between stress, depression, and brain structure. Structural neuroimaging has provided significant evidence on the brain areas that contribute to depressive illness, showing as the most common outcome a reduction in GMV. However, unlike PTSD, in depression there is no univocal relationship between the presence of a stressful event and the onset of depression, although in many cases a precipitating stressful event is identified (especially in the first episodes), this variable is not systematically collected in neuroimaging studies. Therefore, we cannot affirm that structural alterations are a consequence of stress.

### 5.1 Hippocampus

Besides those volume reductions in meta-analysis -in which some of them used ROIS-, original voxel-wise morphometry studies have also found hippocampal smaller GMV in MDD *versus* healthy controls [173]. It has been demonstrated that untreated long depressive episodes were associated with reductions in GMV, suggesting a deleterious impact of MDD progression in brain structure. Childhood maltreatment has also been demonstrated to affect adult hippocampi. A classical study by Vythilingam and colleagues [174], found that the factor explaining smaller hippocampi in MDD participants was childhood abuse instead of MDD diagnosis. More recently, Opel *et al.* [175] reported that hippocampal alterations in MDD patients may at least partly be traced back to a higher incidence of early-life adverse events.

The GMV of this subcortical limbic structure has shown consistent relationships with MDD therapeutic responses. Soriano-Mas *et al.* [176] reported that hippocampal volume decreases predicted a slower recovery after treatment initiation. In this line, a latter meta-analysis [177] confirmed that in MDD patients taking antidepressants, smaller hippocampal volumes predicted lower response/remission rates. Hippocampus has also shown GMV increases induced by ECT, specifically in its anterior part [178]. According to the above and other findings, Fonseka *et al.* [179], set the hippocampus as a reliable therapeutic biomarker of MDD response.

The hippocampus shows high sensitivity to neurobiological processes and many clinical aspects such as illness severity [180], illness later onsets [181] or the absence of comorbid anxiety [182]. Thus, the integrity of its structure is highly related to external stressors -like childhood maltreatment-, antidepressants, or the very illness progression. The capacity of the hippocampal greater volumes to predict better MDD illness trajectories might be indicating a sort of neurobiological resilience to stress.

### 5.2 Amygdala

MDD has traditionally been associated with volumetric alterations in the amygdala. Similarly to the findings in PTSD, the relation between volume and activity within the amygdala is far from being established yet. Studies with functional neuroimaging had consistently found elevated activity in the amygdala in depressed individuals which was associated with symptom severity in the previous decade. However, structural neuroimaging studies yielded variable findings, with some reporting smaller amygdala volumes and some, greater, suggesting no reliable association between depressive disorder and amygdala volumes. Some posterior meta-analyses confirmed that no aggregate-level differentiate between depressed and non-depressed individuals in amygdala volumes due to significant variability among studies [183]. More recent studies are stressing towards the role of the amygdala in MDD by investigating the effects of medication as well as the involvement of specific amygdalar substructures [184, 185].

### 5.3 Prefrontal Cortex

Alterations within the PFC have extensively been reported in the last decades. One of the largest meta-analyses reported in recent years [186] showed smaller GMV relative to healthy controls, mainly in the medial, ventral, and dorsal prefrontal systems, including the ACC. This study, including both MDD and BD patients, shows smaller GMV, smaller GMV in the left parahippocampal gyrus and the right DLPFC -specifically the middle frontal gyrus-. In this regard, voxel-based morphometry (VBM) studies have shown consistent reductions in GMV in the frontal gyrus of MDD patients compared with healthy controls [187-190]. Specifically, reductions in GMV in the superior frontal gyrus have been linked to attentional biases toward negative stimuli, predisposing to possible maladaptive responses to stressful stimuli [191]. In this line, Serra-Blasco *et al.* [192] also described a pronounced reduction of the superior frontal gyrus in patients with long-lasting illnesses. This finding supports the idea of superior frontal gyrus deterioration at advanced illness stages [192]. However, other VBM studies have found significant superior frontal gyrus reductions in patients with first-episode MDD [188, 193], suggesting the possibility of a vulnerability factor rather than an acquired (stress-related) structural alteration.

There is evidence to support the effect of chronic stress on ACC volume, and its relationship to behavioural emotionality. The ACC is highly connected to the “cognitive” PFC and the “emotional” limbic system and, thus, has an important role in affect regulation. Furthermore, it has indeed been identified as a distinctive brain area in MDD psychopathology. More specifically, ACC contributes to some of the abnormalities observed in affective regulation, attention, problem-solving, motivation, and decision-making in patients with depression [194]. Several structural MRI studies have described reductions in GMV in the ACC comparing with healthy controls [195-198]. Results from a study by the ENIGMA MDD Working Group concluded that depression might impact the brain structure differently at different stages of life and described more pronounced thinning cortical grey matter in ACC as well as in posterior cingulate cortex (PCC), orbitofrontal cortex, and temporal lobes in patients with a first depressive episode and adult-onset patients [195]. Furthermore, greater GMV of the dorsal part of the right ACC has been associated with better clinical outcomes, reinforcing the use of structural neuroimaging in conjunction with clinical symptomatology for clinical outcome prediction [199]. In this line, Costafreda *et al.* [200] described that achieving clinical remission after taking antidepressant medication could be predicted from the observation of increased density in the right ACC, among other frontolimbic areas such as the left PCC [200].

For many years, the pivotal role of ACC in major depression eclipsed the relevance of its posterior region of the cingulate cortex. However, since the study of Caetano *et al.* [201], which reported significantly smaller cingulate volumes (including its posterior parts) in MDD patients compared to healthy controls, the evidence of the involvement of PCC in MDD pathophysiology has not stopped rising.

PCC is thought to have a function in focal attention, as it shows a deactivation during attentionally demanding tasks [202]. More specifically, it has been demonstrated that PCC has a main role in internally directed cognition [202]. This hypothesis is partly supported by the discovery of the involvement of PCC in the default mode network [203], in which it shows deactivation during external cognitive processing. Another outstanding cognitive function of PCC is related to episodic memory *via* its direct connections with the hippocampus [204]. Those structural connections have shown differences between HC and MDD patients in structural equation modelling studies [205]. Such differences in organizational patterns might in turn underlie the prominent memory dysfunction observed in MDD patients [206]. Such memory impairments have been shown to be more pronounced as the depressive illness progresses [207, 208], pointing to the potential gradual damage of its underlying structures.

PCC does show consistently smaller grey GMV in MDD patients compared to healthy controls [173, 195, 196]. However, its involvement in MDD pathophysiology seems to be heterogeneous. The study of Shen *et al.* [181], which divided the MDD sample between early-onset and late-onset depression, reported smaller PCC GM only in the early-onset sample. An independent study demonstrated the influence of genetic variants on PCC GMV [209] reporting a reduced PCC GMV only in MDD patients with the T allele carrier of the LHPP gene (rs35936514). These findings could explain the heterogeneity surrounding PCC involvement in MDD across studies.

### 5.4 Other Regions

The insular cortex or insula is involved in a high variety of critical functions altered in neuropsychiatric disorders such as MDD. The roles of the insula range from sensory processing, emotion representation, autonomic control or risk prediction, and decision-making to complex social functions [210]. Such skills are commonly affected in MDD, and thus it is not surprising the amount of evidence that neuroimaging studies have shown supporting its involvement in MDD onset, course, and treatment outcomes.

A number of structural MRI studies have replicated the finding of smaller GMV insular volumes in patients with MDD [173, 186, 211], even in meta-analyses of cortical thickness [195]. Further, more than ten years ago, a VBM study by Soriano-Mas *et al.* [176] showed GMV decreases in the left insula of MDD patients, which were also correlated with slower response to treatment and higher risk of relapse. These findings were shortly confirmed by the study of Serra-Blasco *et al.* [192], which found that longer illness duration was negatively correlated with left insula GMV. Such findings, along with others in the same line, placed the insula as another region of interest with the potential to serve as a biomarker of treatment response [179].

Similar to the other above-mentioned brain regions with close involvement in MDD, the insula has shown relationships with illness duration, depressive symptoms [212], and childhood maltreatment [158]. Also, a recent study of structural covariance has placed the insula at the centre of the three main networks dysfunctional in MDD [213].

One last structure to put focus on is the cerebellum. It has also been the last in gaining a place in the neurobiological bases of stress-related disorders, specifically in MDD, as well as PTSD (previously mentioned). At first, functional involvements were mainly related to movement and did not receive much attention from the scientific community of biological psychiatry, rarely appearing when ROI strategies were employed. Some studies (*i.e*., [214]) and meta-analyses (*i.e*., [215]) mention the cerebellum at the end of their results and not as primary findings, even though this structure displayed the bigger cluster sizes and the most powerful effect-size statistics indicators. Cerebellar reductions are thought to be underneath attentional difficulties and altered emotional responses observed in MDD and other stress-related situations due to the proximitiy of the cerebellum to the limbic system and hypothalamic pituitary adrenal (HPA) axis, both of which play crucial roles in the stress response [172].

Many previous structural MRI studies have found the GMV of the cerebellum diminished in patients with MDD [196, 216, 217] and studies with more homogenous samples have suggested that this structure could get structurally damaged with illness progression [198, 218]. A recent mini-review by Depping and colleagues [219] comprises multidisciplinary evidence demonstrating the involvement of the cerebellum in many aspects of cognition, emotion, and self-referential processing and states its key contribution specifically of its VII lobule (affective/cognitive cerebellum)- to MDD pathophysiology. The review also highlights the neuropsychological role of the VII lobule in the cognitive control network, maybe contributing to the cognitive dysfunction observed in MDD.

## 6 ANXIETY DISORDERS

Anxiety disorders are characterized by excessive and persistent fear and anxiety, avoidance of perceived threats (either external or internal), and significant distress and/or functional impairment.

It is worth noting that, as reflected by recent changes in the main psychiatric diagnostic systems (DSM-5, ICD-11), anxiety disorders have been granted a nosological category independent from other stress-related disorders such as PTSD and MDD. While this classification primarily responds to the need for diagnostic criteria and treatment guidelines in clinical settings [220], current neuroimaging research exploring the neuronal underpinnings of stress-related disorders suggest the existence of alterations specific to anxiety disorders [17, 221]. The category of anxiety disorders includes separation anxiety, selective mutism, specific phobia (SP), social anxiety disorder (SAD), panic disorder (PD), agoraphobia, and generalized anxiety disorder (GAD), each differing in the stimulus and situations inducing maladaptive anxiety responses or, in the case of selective mutism and panic disorder, in its expression. Despite this heterogeneity, several shared constructs and processes are thought to play a role in the etiopathogenesis of the disorders [222], namely uncertain threat anticipation [223], fear excitation, inhibition [224], and generalization [225], hinting at the idea of a common neural circuit responsible for the generalities across disorders.

### 6.1 Hippocampus

This structure has also been included in the models of anxiety disorders due to its function as a modulator of learned fear [226], providing contextual information during threat processing *via* interactions with the amygdala, mPFC, and ACC [227, 228]. Decreased GMV of the hippocampus is frequently reported in GAD [12, 229, 230] but not always [231]. Some studies found this result in PD [232] and SAD but the finding lacked consistency, as shown by recent metanalytical research [233, 234]. No structural alterations in the hippocampus were found in specific phobia research. Therefore, despite the structural vulnerability of the hippocampus to stress processes, the findings of hippocampal atrophy in anxiety disorders are only consistent in GAD. However, recent research suggests that, due to the complexity and functional specificity of hippocampal subnuclei, future efforts will benefit from more precise segmentation methodologies [225, 232].

### 6.2 Amygdala

Early literature exploring the neural underpinnings of anxiety disorders focused on the implication of the amygdaloid complex during emotional or threat processing [235, 236], due to its key role in the neurobiology of fear expression and acquisition [237]. This model proposes that a hyperresponsivity of the amygdala induces lower thresholds in threat detection, increased threat sensitivity, or amplified conditioned responses of fear and anxiety [238], a mechanism inducing and/or maintaining the symptomatology observed in anxiety disorders. This amygdalocentric hypothesis is supported by extensive bibliography regarding structural alterations, however, the results are not homogeneous across disorders. Patients with PD consistently show a reduced volume of the amygdala [239-243]. Interestingly, the opposite pattern has been reported in GAD, where larger amygdala volumes are frequently observed [244] but not always [245, 246]. For SAD [247] and SP [248-251], amygdalar GM alterations are not consistently observed. To our knowledge, there is no published MRI research assessing structural brain alterations in selective mutism, separation anxiety disorder, or agoraphobia without panic disorder.

Therefore, current research indicates volumetric alterations of the amygdala are specifically related to panic disorder, where reductions in the lateral and basal nuclei are theorized to cause, respectively, a misjudgment of both sensory information (mediated by the amygdala) and orbitofrontal cortex risk assessment (mediated by the orbitofrontal cortex) that contributes to panic attacks [241]. Another possible interpretation of this result is that panic disorder, due to its episodes of acute fear, has a higher allostatic load within the hub of the fear circuit compared to the other anxiety disorders [228]. Consequently, the discrepancy with GAD results of increased volume could be explained by the different impacts of acute and chronic stress in brain morphology, the latter being associated with increased dendritic growth in the amygdala [64, 252]. However, recent meta-analytic studies addressed to determine shared structural alterations across anxiety disorders do not include the results in the amygdala, probably due to the heterogeneity among anxiety disorders [17, 253].

### 6.3 Prefrontal Cortex

More recent models describing the neurocircuitry of anxiety disorders acknowledge the role of emotion modulation regions, with a special interest in the mPFC [254] but also including the hippocampus, the DLPFC, the ventrolateral PFC, the orbitofrontal cortex, and the ACC [255, 256]. The mPFC has been defined as a main node of top-down regulation of the amygdala [257, 258], a function thought to be compromised in anxiety disorders [259] due to an underperforming frontal function [260, 261]. In agreement, midline prefrontal atrophy, including ACC and mPFC, has been suggested as a structural alteration in transdiagnostic anxiety, strongly supported by meta-analytic results [17, 253, 262]. When reviewing published results for each disorder, results of diminished volumes in mPFC and ACC were fairly consistent in GAD [229, 231, 246, 263, 264], however, it is worth noting no frontal lobe alterations were found in the larger mega-analysis assessing thickness and surface area metrics [265]. Prior research has indicated that PD patients might also suffer from this frontal reduced volumes [240, 266, 267] yet more recent works have failed to replicate results [233, 243, 268]. While some mixed results emerged from social anxiety disorder [269, 270], most results do not support the hypothesis of structural frontal alterations [233, 234, 271]. Among the limited research in specific phobia, a subtype- specific effect is suggested as results indicate increased mPFC and ACC volumes in dental phobia [249] and reduced in spider phobia [250] yet more research is required to reach a conclusive answer. Overall, a consistent body of evidence indicates there is a reduction in frontal GMV, mainly of the mPFC and the ACC, in GAD and, to a lesser degree, in PD. Said alteration is thought to be linked to deficient top-down emotional regulation, with recent meta-analytic research suggesting volume reductions in mPFC [253] and ACC [17] as candidates of common alterations across anxiety disorders and MDD.

### 6.4 Other Regions

While outside the frontolimbic pathway, the insula is a heavily interconnected hub with communication with cortical and subcortical regions involved in emotional, cognitive, and motivational functions, as described before. The critical role in interoception and control of autonomic function is also implicated as well as in emotional processing, specifically, in fear and anxiety [210]. The more consistent results indicate structural alterations emerged in PD, where patients presented reduced volumes in the insula as one of the most robust findings of structural alteration in the disorder [233, 234, 242, 272]. A similar result has also been found in GAD literature [230, 231] yet less frequently replicated in comparison to structural abnormalities within frontal regions [246, 264]. Recent research has suggested that insular atrophy might be a consequence of long illness duration [231]. Volumetric reductions of the insula have been found in SAD [273-275], although results come mainly from research using a priori ROI analyses and are not replicated by more recent whole-brain analyses and metanalyses [233, 234, 270]. Two articles have found structural alterations in the insula in specific phobia [249, 251], with reduced volume in dental phobia and increased cortical thickness in animal phobia, highlighting the insula as a region of interest for future SP research.

As with the insula, the striatum is also included in the emotion-generating and processing regions within proposed neurocircuitry models of anxiety [256], being key in motivational processes and learning and with connections with limbic structures [276]. Interestingly, volume reductions in the putamen are being highlighted as the main alteration observable in SAD by the most recent metanalysis [234, 277], an alteration that presents certain specificity to SAD. Consequently, while some papers reported decreased volumes in the putamen [278] and the nucleus accumbens [279] in patients with PD, results outside SAD are not consistent.

Despite not being in the spotlight of current models, regions implicated in the processing of sensorial information are suggested to play a certain role in anxiety disorders [256]. Decreased thalamic volumes have been reported mainly in SAD [234, 277, 280] but also in GAD [230] and PD [281]. Conversely, increased volumes in the fusiform gyrus [282, 283] have been found in SAD, whereas, signs of grey matter reductions have been found in PD [268]. While relatively scarce, research finding structural alterations within said sensory processing regions suggest a special significance for patients with SAD, consistent with the key role of the fusiform gyrus in face processing and social anxiety [284].

## 7 DISCUSSION

Convergent evidence from structural neuroimaging studies has demonstrated the overlapping of volumetric brain alterations on stress-related disorders. These studies might suggest shared biological underpinnings that go beyond diagnostic boundaries, which coincide with the rationale behind the Research Domain Criteria (RDOC) initiative whose goals were to develop “new ways of classifying psychopathology based on dimensions of observable behavior and neurobiological measures” by incorporating basic dimensions of functioning to be studied across multiple levels of analysis, from genes to neural circuits to behaviors, “cutting across disorders as traditionally defined” [285]. Neuroimaging studies have become essential to fit in such a bottom-up approach and should have facilitated the goal of an etiology-based nosology. However, as seen in the preceding sections, many aspects need to be further explored, as there are indeed shared fronto-limbic alterations across stress-related disorders, but also, some particular brain regions seem to be specifically affected or preserved in each psychiatric condition encompassed within stress-related disorders.

Fig. (**1**) summarizes the brain regions identified in stress-related disorders. As mentioned above, the identification of several of these regions, such as the hippocampus, amygdala, or ACC, is mostly based on hypothesis-driven studies or regions of interest. Although these studies are widely used and hold notable scientific value, they are explicitly characterized by neglecting other brain regions, also likely to provide the specificity required for the development of personalized diagnostic, predictive and therapeutic biomarkers. A detailed summary of brain regions identified in structural magnetic resonance imaging (MRI) studies focused on the main stress-related disorders, including PTSD, MDD and anxiety disorders, is presented in Tables **1**-**3**, respectively, to provide a more comprehensive overview of the regions detected.

Genetic studies have documented familial coaggregation and co-heritability between stress-related disorders and other psychiatric phenotypes [9]. Family and twin studies have shown cross-disorder familial risk and high degrees of genetic correlation among PTSD, MDD, and anxiety disorders, supporting the phenomenon of pleiotropy that has been identified for common and rare SNVs and rare CNVs and other structural variants in neuropsychiatric disorders, which are frequently overrepresented in the fronto-limbic regions reported so far [286]. While genetic influences on structural brain characteristics of stress-related disorders overlap to some range, the biological basis of this overhang is still unknown. Moreover, the available literature is fairly filling this gap of knowledge by the investigation at multiple levels, which should include pleiotropic effects (in cellular and animal models) to human clinical neuroscience (*e.g*., neuroimaging), and is up to now a great challenge.

One of the main difficulties is to disentangle whether the structural brain differences detected in stress-related disorders are, in essence, stress-related, or disease-related. While in PTSD the relationship with traumatic stress and stress-based fear conditioning responses is undeniable, in other disorders the presence of stress, although crucial, is not systematically documented or identified. One could speculate about the causal effects of stress in the development and display of stress-related disorders. However, the most parsimonious view, based on the available literature, is a bidirectional relation in the sense of a continuum between structural brain impairments due to stress events and the presence of a stress-related disorder. Neuroimaging findings do not allow us to know whether exposure to acute or chronic stress is a necessary and sufficient condition for all the encompassed stress-related disorders. While causality seems clear for PTSD, this is not the case for MDD or anxiety disorders. Therefore, when interpreting structural brain changes in stress-related disorders, three possible scenarios emerge: a) such volumetric changes within particular brain structures are acquired, *i.e*. a consequence of exposure to stress, and thus, these could arise in any individual exposed to a stressful situation; b) these changes only appear in individuals who develop the disease and thus are disease-specific, and when exposure to stress can be confirmed, they could be disease- and stress-specific; and c) these alterations are pre-existing, so it can be assumed that they are inherited and constitute a predisposing or vulnerability factor.

This means that evidence of how stress impacts brain development and confers risk for the future development of pathology is still at the early stages. To solve some of these issues, future neuroimaging studies should include detailed measures of stress in early and adult life, valid proxies of cumulative stress to be associated with the disease as well as markers of the deleterious effect of the disorder itself to identify similar associations as the ones observed in PTSD. However, this is far to be a realistic pathway to reach such an ambitious goal for two reasons: exposure to acute or chronic stressful events does not always end up with the development of a psychiatric condition (not even PTSD), and some patients who develop stress-related disorders (even PTSD) may have been exposed to a not-so-stressful situation, but subjectively perceived as so. Multi-level factors should be taken into account such as subjective measures of stress, personality characteristics, cope styles, *etc*. Therefore, in pursuit of defining the putative causal relationship between stress and psychopathology, a breakthrough is needed, which comes from a deep review of what is done to date, and what would be the new ventures able to encompass the multifactorial nature of the pathophysiology of stress-related disorders.

The intricate interplay of genetic and environmental factors -in the wider sense- is part of the endeavor to understand the pathophysiology of mental disorders, and in particular stress-related disorders. Epigenetic studies have represented a promising breakthrough in this regard. Environmental influences such as early and lifetime stress exposure have been found to shape epigenetic patterns, altering the function of the genome across the lifespan. At the same time, new evidence have shed light on how treatment -both pharmacological and psychotherapeutic as potential beneficial environmental factors- can change and revert epigenetic risk patterns [287]. Moreover, it is well-established that epigenetic changes induced by stressors persist over time, could be passed down to the next generations [288, 289], and could play a mediating role in shaping brain structures through glucocorticoid signaling pathways -for instance, altered transcriptome regulation caused by epigenetic changes in glucocorticoid-related genes had already been associated with the development of stress-related disorders- [290]. If confirmed, structural brain changes could become markers for treatment interventions, *i.e*., defining personalized strategies depending on detectable long-lasting epigenetic changes that embed observable shape characteristics of key brain structures. But again, multiple variables may account for individual differences that should be taken into consideration.

As reviewed in prior sections, results of structural abnormalities differ across stress-related disorders. Recent research has employed meta-analytic analyses or transdiagnostic samples in the search for a shared pattern across a host of these diagnostics in which stress seems to be either a necessary or facilitating condition. Decreases in grey matter within the hippocampus, the prefrontal cortex, and other regions (*e.g*., insula, the superior temporal gyrus, or the cerebellum) have been reported across disorders yet with conflicting results regarding whether these reductions are truly shared among the variety of stress-related disorder. The question that emerges from these findings is how many factors may underlie these group differences which could be determinant for each individual. Mega-analysis methods [291] are now used to evaluate the specificity of structural alterations in stress-related disorders and hopefully will help reveal the potential relationship with core dimensions of psychopathology, rather than hermetic categories. In this regard, the current classification of disorders in the DSM-5 is a sign of the times, and in a few years, it will be clear the utility of deleting the exclusion of stressors as possible causes of a depressive episode, the use of stressors as specifiers in anxiety disorders or the appearance of a new diagnose category -acute stress disorder- facilitate tailoring of diagnoses and treatments. In this regard, new pharmacological strategies such as ketamine, esketamine, and other antidepressant-like NMDA receptor modulators, have been linked with the pathogenesis of stress-related disorders and might have beneficial effects in preclinical and clinical studies for PTSD [292] MDD [293, 294] and anxiety disorders [295].

## CONCLUSION

While common and distinct structural alterations in key brain regions have been consistently identified in the different stress-related disorders, there are still unanswered questions. The occurrence of a traumatic stressful event is only a prerequisite for establishing the diagnosis of PTSD; in all other stress-related pathologies, although stress is a crucial pathogenic factor, it is not always systematically measured, examined, or reported. Extensive longitudinal studies are needed to establish the relationship of stress more comprehensively and unequivocally to changes in brain structure throughout the disease. Thus, it may be time for stress research to move away from the classical approach and adopt new approaches to capture the multifaceted nature of stress. The intricate relationship between the effects of stress, disease burden, neurodevelopmental stage, genetic vulnerability, personality traits, or cognitive styles can only be accurately unravelled from appropriately designed multimodal and collaborative studies. This is a prerequisite for translating the wealth of knowledge generated in the research arena to real-world clinical practice.

## Figures and Tables

**Fig. (1):**
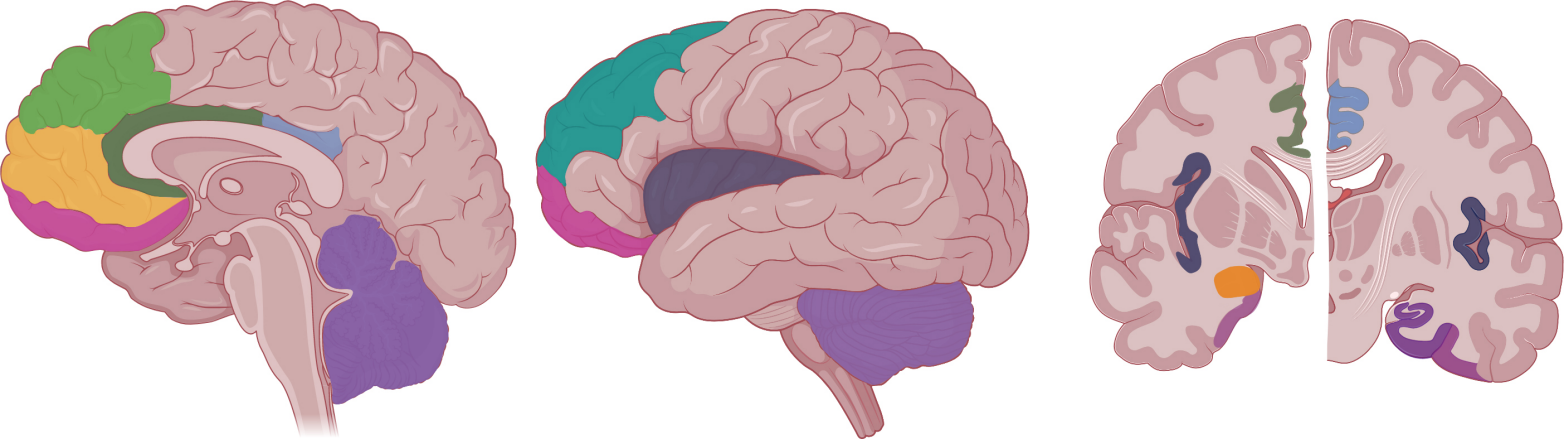
Brain regions prominent in the literature of stress-related disorders: the dorsomedial prefrontal cortex (dmPFC; light green), ventromedial prefrontal cortex (vmPFC; light orange), orbitofrontal cortex (OFC; magenta), anterior cingulate gyrus (ACC; dark green), posterior cingulate gyrus (PCC; light purple), cerebellum (grape), dorsolateral prefrontal cortex (dlPFC; turquoise green), insula (dark gray), amygdala (dark orange), hippocampus (plum) and parahippocampus (heliotrope). Created with BioRender.com.

**Table 1:** Brain morphological alterations in post-traumatic stress disorder.

**Study**	**Participants**	**Analysis**		**Brain Results**
Nardo *et al*., 2010 [167]	21 PTSD/22 TC	Whole-brain VBM	**↓**	GMC - PCG, Precuneus Cortex, Lingual Gyrus, Parahippocampus
Zhang *et al*., 2011 [169]	10 PTSD/10 TC	Whole-brain VBM	**↓**	GMV - Hippocampus, Parahippocampus, Calcarine Cortex
Baldaçara *et al*., 2012 [172]	42 PTSD/42 TC	ROI VBM: Cerebellum	**↓**	GMV - Cerebellum
Tavanti *et al*., 2012 [165]	25 PTSD/25 HC	Whole-brain VBM	**↓**	GMV - Frontal Pole, SFG, MFG, IFG-Pars Triangularis, Paracingulate Gyrus, Precentral Gyrus, Postcentral Gyrus, PCG, Intracalcarine Cortex, Lateral Occipital Cortex, Lingual Gyrus, Occipital Fusiform Gyrus, Occipital Pole, Precuneus Cortex, Supracalcarine Cortex, Temporal Occipital Fusiform Cortex, ITG
Tan *et al*., 2013 [168]	12 PTSD/14 TC	Whole-brain VBM	**↓**	GMV - Superior Parietal Lobule, SFG
Bossini *et al*., 2017 [166]	19 PTSD/19 HC	Whole-brain VBM	**↓** **↑**	GMV - Parahippocampus, SMA, Lingual Gyrus, SFG GMV - Angular Gyrus, Inferior Parietal Lobe, ITG

**Abbreviations:** *GMC,* Gray Matter Concentration; *GMV,* Gray Matter Volume; *HC*, Healthy Controls; *IFG*, Inferior Frontal Gyrus; *ITG*, Inferior Temporal Gyrus; *MFG*, Middle Frontal Gyrus; *PCG*, Posterior Cingulate Gyrus; *PTSD*, Post-Traumatic Stress Disorder; *ROI*, Region-of-Interest; *SFG*, Superior Frontal Gyrus; *SMA*, Supplementary Motor Area; *TC*, Traumatized Controls; *VMB*, Voxel-Based Morphometry.

**↓** Lower regional GMV/GMC in patients.

**↑** Greater regional GMV/GMC in patients.

**Table 2:** Brain morphological alterations in major depressive disorder.

**Study**	**Participants**	**Analysis**		**Brain Results**
Vasic *et al*., 2008 [190]	15 MDD/14 HC	Whole-brain VBM	**↓**	*GMC - IFG, ITG, OFC, MeFG, Transverse Temporal Gyrus* *GMV - Thalamus, Hippocampus, Cingulate Gyrus*
Leung *et al*., 2009 [191]	17 MDD/17 HC	Whole-brain VBM	**↓**	*GMC - SFG, MFG, MeFG, OFC, ACC, MCC, Insula, Precentral Gyrus, Fusiform Gyrus* *GMV - MCC, Precuneus, Angular Gyrus, MTG, Temporal Pole, Precentral Gyrus* *GMV - MFG, PCG*
Abe *et al*., 2010 [189]	21 MDD/42 HC	Whole-brain VBM	**↓**	GMV - Parahippocampus, Hippocampus, MFG, ACC, Parietal Lobe, Occipital Lobe, STG
Salvadore *et al*., 2011 [197]	58 dMDD / 27 rMDD / 107 HC	Whole-brain VBM	**↓** **↑** **↓** **↑**	GMD (*vs*. dMDD) - SFG, MFG, SFG GMD (vs. rMDD) - Putamen, Insula, STG, IFG, IPL, SPL, Postcentral Gyrus GMV (*vs*. dMDD) - IFG, SFG, MFG GMV (*vs*. dMDD) - Subgenual ACC, Pregenual ACC
Soriano-Mas *et al*., 2011 [176]	70 MDD / 40 HC	Whole-brain VBM	**↓**	GMV - Insula
Grieve *et al*., 2013 [196]	102 MDD/ 34 HC	Whole-brain VBM	**↓**	GMV - MFG, IFG, Rectal gyrus, SFG, MFG, ACC, MCC, PCC, Precuneus, Precentral gyrus, Fusiform Gyrus, ITG, MTG, STG, Postcentral gyrus, IP, Cuneus, MOG, IOG, Thalamus, GP, Caudate, Cerebellum
Serra-Blasco *et al*., 2013 [192]	22TRD/22REMREC/22FIRST/32 HC	Whole-brain VBM ROI SBM (volume): ACC, SFG, MeFG, Insula	**↓**	GMV (TRD<HP) - SFG, ACC, MeFG, Insula, IFG, Parahippocampus, Transverse-Temporal Gyrus, Post-Central Gyrus *GMV (REM-REC<HP) - SFG, Cerebellum, ACC* *GMV (TRD<FIRST) - Pre-Central Gyrus, Post-Central Gyrus, MeFG, Insula, Transverse-Temporal Gyrus, Inferior Parietal Lobule, PCG* *Volume (TRD<FIRST) - MeFG, Insula* *Volume (TRD<REM-REC) - MeFG*
Jung *et al*., 2014 [187]	50 MDD/29 HC	Whole-brain VBM	**↓**	*GMV - Insula, ACC, SFG, IFG, OFC, Amygdala*
Lai & Wu, 2014 [188]	38 MDD/27 HC	Whole-brain VBM	**↓**	GMV - SFG, MFG, MeFG, Insula
Machino *et al*., 2014 [198]	29 MDD / 29 HC	Whole-brain VBM	**↓**	GMV - Dorsal ACC, Ventral ACC, SFG, and Cerebellum
Tae, 2015 [173]	20 MDD/21 HC	Whole-brain VBM	**↓**	GMV - Amygdala, Hippocampus, Parahippocampus, Thalamus, PCG, Pregenual Gyrus, Lingual Gyrus, Insula, Fusiform Gyrus, IFG
Shen *et al*., 2016 [181]	147 MDD / 130 HC	Whole-brain VBM	**↓**	*GMV - Precuneus, Hippocampus, MTG, Cerebellum*
Yang *et al*., 2017 [214]	35 MDD / 23 HC	Whole-brain VBM	**↓**	GMV - *Insula, Putamen, Amygdala, Lingual Gyrus,* and *Cerebellum*
Zhao *et al*., 2017 [211]	37 MDD / 24 SAD / 41 HC	Whole-brain VBM	**↑** **↓**	GMV - Temporal Pole GMV - OFC, Putamen, Thalamus, MFC, Cuneus
Han *et al*., 2021 [218]	195 MDD / 130 HC	Whole-brain VBM	**↓** **↑**	GMV - Thalamus, Parahippocampal Gyrus, Lingual Gyrus, Hippocampus, Fusiform Gyrus, and Cerebellum GMV - VeMPFC, Amygdala, Precuneus, Postcentral Gyrus
Kandilarova *et al*., 2019 [194]	39 MDD/ 42 HC	Whole-brain VBM	**↓**	GMV - MeFC, ACC, MTG, IFG (pars triangularis)
Lee *et al*., 2011 [217]	47 MDD / 51 HC	Whole-brain VBM	**↓**	GMC - Midbrain (dorsal raphe nuclei, DRN), Subgenual ACC, Thalamus, Insula, Accu, Amygdala, Hippocampus, Fusiform Gyrus, Lingual Gyrus, Lingual Gyrus, MTG, STG, and Cerebellum
Peng *et al*., 2011 [216]	22 MDD / 30 HC	Whole-brain VBM	**↓**	*GMD - STP, Insula, IFG, MTG, STG, OFG, Parahippocampal Gyrus, MFG,* and *Cerebellum*
Zhang *et al*., 2021 [212]	30 MDD / 63 HC	Whole-brain VBM	**↓**	GMV - Insula, STP, MTP, Olfactory Gyrus, Gyrus Rectus, IOFG, Putamen, Caudate, STG, Cerebellum, Lingual Gyri, Parahippocampal Gyrus, Fusiform Gyri, ITG

**Abbreviations:** ACC, Anterior Cingulate Cortex; Accu, Nucleus Accumbens; dMDD, currently depressed MDD patients; DALPFC dorsal anterolateral prefrontal cortex; DMPFC, dorsomedial prefrontal cortex; *FIRST*, First-Episode; GMC, Gray Matter Concentration; GMV, Gray Matter Volume; GP, Globus Pallidus; HC, Healthy Controls; IFG, Inferior Frontal Gyrus; ITG, Inferior Temporal Gyrus; IP, Inferior Parietal; IOFG, Inferior Orbitofrontal Gyrus; LOFC, Lateral Orbitofrontal Gyrus; MeOFC, Medial Orbitofrontal Gyrus; MCC, MidCingulate Cortex; MDD, Major Depression Disorder; MeFG, Medial Frontal Gyrus; MeFC Medial Frontal Cortex; MFG, Middle Frontal Gyrus; MTG, Middle Temporal Gyrus; MTP, Middle temporal Pole; MOG, Middle Occipital Gyrus; NA, Not Available; IOG, IPL, Inferior Parietal Lobule; Inferior Occipital Gyrus; OFC, Orbitofrontal Cortex; OFG, Orbitofrontal Gyrus; PCG, Posterior Cingulate Gyrus; PCC, Posterior Cingulate Cortex; *REM-REC*, Remitted-Recurrent; *ROI*, Region-of-Interest; SBM, Surface-Based Morphometry; SFG, Superior Frontal Gyrus; STP, Superior Temporal Pole; SPG, Superior Parietal Gyrus; SPL, Superior Parietal Lobule; Superior Parietal Lobule; rMDD, currently remitted MDD patients; STG, Superior Temporal Gyrus; *TRD*, Treatment-Resistant Depression; VLPFC, Ventrolateral Prefrontal Cortex; VeMPFC, Ventral Medial Prefrontal Cortex; *VBM,* Voxel-Based Morphometry.

**↓** Lower regional GMV/GMC in patients.

**↑** Greater regional GMV/GMC in patients.

*Italics* = Uncorrected Brain Results (or corrected with *p*>.001 AlphaSim).

**Table 3:** Brain morphological alterations in anxiety disorders.

**Study**	**Participants**	**Analysis**		**Brain Results**
Massana *et al*., 2003 [239]	12 PD / 12 HC	ROIs VBM: amygdala, hippocampus, temporal lobe	**↓**	GMV - Amygdala
Rauch *et al*., 2004 [251]	10 SP animal / 20 HC	ROIs^1^ SBM(t)	**↑**	SBM(t) - Insula, pregenual ACC, PCC, superior and medial occipital cortex
Yoo *et al*., 2005 [278]	18 PD / 18 HC	Whole-brain VBM	**↓**	GMV - putamen
Uchida *et al*., 2008 [267]	19 PD / 20 HC	ROIs^2^ VBM	**↑** **↓**	*GMV - insula, midbrain* and *pons* *GMV - ACC*
Asami *et al*., 2009 [240]	24 PD / 24 HC	Whole-brain VBM	**↓**	GMV - dmPFC, vmPFC, amygdala, ACC, STG, insula, occipitotemporal, cerebellum
Hayano *et al*., 2009 [242]	30 PD / 30 HC	ROIs VBM: amygdala, hippocampus	**↓**	GMV - Amygdala
Liao *et al*., 2011 [269]	18 PD / 18 HC	Whole-brain VBM	**↑** **↓**	GMV - mPFC GMV - ITG, hippocampus, parahippocampus
Hettema *et al*., 2012 [12]	17 GAD / 17 HC	ROIs VBM: amygdala, hippocampus, ACC, OFC	**↓**	*GMV - Hippocampus*
Syal *et al*., 2012 [275]	13 SAD / 13 HC	Whole-brain SBM(t,v)	**↓**	SBMt - Fusiform gyrus, ITG, Frontal and Temporal poles, mOFC, Insula, Postcentral and Supramarginal
Talati *et al*., 2012 [282]	33 SAD/ 16 PD/ 37 HC	Whole-brain VBM	**↑** **↓** **↑** **↓**	GMV SAD - Fusiform, parahippocampus, cerebellum GMV SAD - STG GMV PD - Cuneus GMV PC - Precentral, postcentral, MCC
Fisler *et al*., 2013 [248]	20 SP (spiders) / 20 HC	ROI VBM amygdala	**↓**	GMV - Amygdala
Na *et al*., 2013 [266]	22 PD (12 with agoraphobia) / 22 HC	ROIs^3^ VBM amygdala, hippocampus, parahippocampus thalamus, insula	**↓**	*GMV PD with agoraphobia - mOFC*
Linares *et al*., 2014 [250]	19 SP (spiders)/ 17 HC	Whole-brain SBM(t)	**↓**	SBMt - ACC
Lai *et al*., 2015 [272]	53 PD / 54 HC	Whole-brain VBM	**↓**	GMV - IFG, insula
Tükel *et al*., 2015 [283]	27 SAD / 27 HC	Whole-brain VBM	**↑**	GMV - MTG, ITG, Precuneus, Fusiform
Moon *et al*., 2017 [230]	20 GAD / 20 HC	Whole-brain VBM	**↓**	*GMV - Midbrain, Thalamus, Hippocampus, Insula, STG*
Makovac *et al*., 2016 [245]	19 GAD / 19 HC	Whole-brain VBM	**↓**	GMV - Supramarginal, postcentral, precentral
Kawaguchi *et al*., 2016 [274]	13 SAD / 18 HC	ROI VBM: Insula	**↓**	GMV - Insula
Andreescu *et al*., 2017 [264]	28 GAD / 31 HC	Whole-brain SBM(t,v)	**↓**	*SBMt - mOFC, rACC* *SBMv - IFG*
Asami *et al*., 2018 [241]	38 PD / 38 HC	ROI SBM(v): Amygdala	**↓**	SBMv - Amygdala
Bas-Hoogendam *et al*., 2018 [247]	39 SAD (22 subclinic) / 62 Hc	ROIs^4^ SBM(t,v,a)	**↓** **↓**	SBMt - rACC, inferior parietal, supramarginal, temporal pole, transverse temporal SBMt - MFG, OFG, STG, Fusiform, rACC
Asami *et al*., 2018b [281]	25 PD / 25 HC	ROI VBM and SBMs: Thalamus	**↓**	GMV - Thalamus SBMs inward deformation
Ma *et al*., 2019 [246]	27 GAD / 28 HC	Whole-brain VBM	**↓**	GMV - Precentral gyrus, SFG
Chen *et al*., 2020 [231]	72 GAD / 57 HC	Whole-brain VBM	**↓**	GMV - IFG, sgACC, vmPFC, dmPFC, insula
Kunas *et al*., 2020 [243]	143 PD / 178 HC	ROIs^5^ VBM	**↓**	GMV - Amygdala
Li *et al*., 2020 [263]	19 GAD / 19 HC	ROIs VBM: ACC, insula, SMA, PFC	**↓**	*GMV - mPFC, ACC, OFC*
Ni *et al*., 2020 [268]	26 PD / 27 HC	Whole-brain SBM(t)	**↓**	SBMt - Fusiform
Zhang *et al*., 2020 [270]	32 SAD / 32 HC	Whole-brain SBM(t,a)	**↓** **↑**	SBMt - SFG, MFG, rACC SBMa - SFG rACC MFG STG OFC
Yoshida *et al*., 2020 [279]	38 PD / 38 HC	ROI SBM(v,s): NAcc	**↓** **↑**	SBMv - NAcc SBMs - NAcc inward deformation
Takaishi *et al*., 2021 [232]	38 PD / 38 HC	ROIs SBM(v): Hippocampal subfields	**↓**	SBMv - Hippocampus (cornu ammonis 2/3)
Atmaca *et al*., 2021 [273]	21 SAD / 20 HC	ROI VBM: insula	**↓**	GMV - insula
Zhang *et al*., 2022 [277]	49 SAD / 53 HC	Whole-brain VBM	**↓**	GMV - thalamus, putamen, parahippocampus

**Abbreviations:** *ACC*, Anterior Cingulate Cortex; *dmPFC,* dorsomedial Prefrontal Cortex; *GAD*, Generalized Anxiety Disorder; *GMV*, Gray Matter Volume; *HC*, Healthy Controls; *IFG*, Inferior Frontal Gyrus; *ITG*, Inferior Temporal Gyrus; *MCC*, Middle Cingulate Cortex; *MFG*, Middle Frontal Gyrus; *MTG*, Middle Temporal Gyrus; *NAcc*, Nucleus Accumbens; *OFC*, Orbitofrontal Cortex; *PCC*, Posterior Cingulate Cortex*; PD,* Panic Disorder; *rACC*, rostral Anterior Cingulate; *ROI*, Region-of-Interest*; SAD*, Social Anxiety Disorder; *SBM(t,v,a,s)*, Surface-Based Morphometry (thickness, volume, area, shape); *sgACC*, subgenual Anterior Cingulate; *SMA*, Supplementary Motor Area; *SP*, specific phobia; *STG*, Superior Temporal Gyrus*; vmPFC, ventromedial Prefrontal Cortex.*

^1^: OFC, cingulate cortex, insula, parahippocampus, temporal pole, somatosensory cortex, visual cortex.

^2^: Amygdala, hippocampus, insula, ACC, thalamus, hypothalamus, midbrain.

^3^: Amygdala, hippocampus, parahippocampus thalamus, insula, mOFC.

^4^: Amygdala, hippocampus, pallidum, putamen, SFG, MFG, OFG, precentral gyrus, cACC, rACC, insula, superior parietal, inferior parietal, precuneus, supramarginal gyrus, postcentral gyrus, temporal pole, ITG, STG, fusiform gyrus and transverse temporal gyrus.

^5^: Amygdala, ACC, hippocampus, insula, thalamus, OFC.

**↓** Lower regional GMV/SBM in patients.

**↑** Greater regional GMV/SBM in patients.

*Italics* = Uncorrected Brain Results (or corrected with *p*>.001 AlphaSim).

## LIST OF ABBREVIATIONS

ACC: Anterior Cingulate Cortex
BDNF: Brain-derived Neurotrophic Factor
BLA: Basolateral Amygdala
DTI: Diffusion Tensor Imaging
GAD: Generalized Anxiety Disorder
GM: Gray Matter
HPA: Hypothalamic Pituitary Adrenal
MDD: Major Depressive Disorder
mPFC: Medial Prefrontal Cortex
MRI: Magnetic Resonance Imaging
PD: Panic Disorder
PFC: Prefrontal Cortex
PTSD: Post-traumatic Stress Disorder
SAD: Social Anxiety Disorder
STG: Superior Temporal Gyrus
TrkB: Tyrosine Receptor Kinase B
VBM: Voxel-based Morphometry

## References

[1] Kessler R.C., Aguilar-Gaxiola S., Alonso J., Chatterji S., Lee S., Ormel J., Üstün T.B., Wang P.S. (2009). The global burden of mental disorders: An update from the WHO World Mental Health (WMH) Surveys.. Epidemiol. Psichiatr. Soc..

[2] (2022). GBD 2019 Mental Disorders Collaborators. Global, regional, and national burden of 12 mental disorders in 204 countries and territories, 1990-2019: a systematic analysis for the Global Burden of Disease Study 2019.. Lancet Psychiatry.

[3] Kessler R.C., Aguilar-Gaxiola S., Alonso J., Benjet C., Bromet E.J., Cardoso G. (2017). Trauma and PTSD in the WHO world mental health surveys.. Eur. J. Psychotraumatol..

[4] Vos T., Allen C., Arora M., Barber R.M., Bhutta Z.A., Brown A., Carter A., Casey D.C., Charlson F.J., Chen A.Z., Coggeshall M., Cornaby L., Dandona L., Dicker D.J., Dilegge T., Erskine H.E., Ferrari A.J., Fitzmaurice C., Fleming T., Forouzanfar M.H., Fullman N., Gething P.W., Goldberg E.M., Graetz N., Haagsma J.A., Hay S.I., Johnson C.O., Kassebaum N.J., Kawashima T., Kemmer L., Khalil I.A., Kinfu Y., Kyu H.H., Leung J., Liang X., Lim S.S., Lopez A.D., Lozano R., Marczak L., Mensah G.A., Mokdad A.H., Naghavi M., Nguyen G., Nsoesie E., Olsen H., Pigott D.M., Pinho C., Rankin Z., Reinig N., Salomon J.A., Sandar L., Smith A., Stanaway J., Steiner C., Teeple S., Thomas B.A., Troeger C., Wagner J.A., Wang H., Wanga V., Whiteford H.A., Zoeckler L., Abajobir A.A., Abate K.H., Abbafati C., Abbas K.M., Abd-Allah F., Abraham B., Abubakar I., Abu-Raddad L.J., Abu-Rmeileh N.M.E., Ackerman I.N., Adebiyi A.O., Ademi Z., Adou A.K., Afanvi K.A., Agardh E.E., Agarwal A., Kiadaliri A.A., Ahmadieh H., Ajala O.N., Akinyemi R.O., Akseer N., Al-Aly Z., Alam K., Alam N.K.M., Aldhahri S.F., Alegretti M.A., Alemu Z.A., Alexander L.T., Alhabib S., Ali R., Alkerwi A., Alla F., Allebeck P., Al-Raddadi R., Alsharif U., Altirkawi K.A., Alvis-Guzman N., Amare A.T., Amberbir A., Amini H., Ammar W., Amrock S.M., Andersen H.H., Anderson G.M., Anderson B.O., Antonio C.A.T., Aregay A.F., Ärnlöv J., Artaman A., Asayesh H., Assadi R., Atique S., Avokpaho E.F.G.A., Awasthi A., Quintanilla B.P.A., Azzopardi P., Bacha U., Badawi A., Balakrishnan K., Banerjee A., Barac A., Barker-Collo S.L., Bärnighausen T., Barregard L., Barrero L.H., Basu A., Bazargan-Hejazi S., Beghi E., Bell B., Bell M.L., Bennett D.A., Bensenor I.M., Benzian H., Berhane A., Bernabé E., Betsu B.D., Beyene A.S., Bhala N., Bhatt S., Biadgilign S., Bienhoff K., Bikbov B., Biryukov S., Bisanzio D., Bjertness E., Blore J., Borschmann R., Boufous S., Brainin M., Brazinova A., Breitborde N.J.K., Brown J., Buchbinder R., Buckle G.C., Butt Z.A., Calabria B., Campos-Nonato I.R., Campuzano J.C., Carabin H., Cárdenas R., Carpenter D.O., Carrero J.J., Castañeda-Orjuela C.A., Rivas J.C., Catalá-López F., Chang J-C., Chiang P.P-C., Chibueze C.E., Chisumpa V.H., Choi J-Y.J., Chowdhury R., Christensen H., Christopher D.J., Ciobanu L.G., Cirillo M., Coates M.M., Colquhoun S.M., Cooper C., Cortinovis M., Crump J.A., Damtew S.A., Dandona R., Daoud F., Dargan P.I. (2016). das Neves, J.; Davey, G.; Davis, A.C.; Leo, D.D.; Degenhardt, L.; Gobbo, L.C.D.; Dellavalle, R.P.; Deribe, K.; Deribew, A.; Derrett, S.; Jarlais, D.C.D.; Dharmaratne, S.D.; Dhillon, P.K.; Diaz-Torné, C.; Ding, E.L.; Driscoll, T.R.; Duan, L.; Dubey, M.; Duncan, B.B.; Ebrahimi, H.; Ellenbogen, R.G.; Elyazar, I.; Endres, M.; Endries, A.Y.; Ermakov, S.P.; Eshrati, B.; Estep, K.; Farid, T.A.; Farinha, C.S.S.; Faro, A.; Farvid, M.S.; Farzadfar, F.; Feigin, V.L.; Felson, D.T.; Fereshtehnejad, S-M.; Fernandes, J.G.; Fernandes, J.C.; Fischer, F.; Fitchett, J.R.A.; Foreman, K.; Fowkes, F.G.R.; Fox, J.; Franklin, R.C.; Friedman, J.; Frostad, J.; Fürst, T.; Futran, N.D.; Gabbe, B.; Ganguly, P.; Gankpé, F.G.; Gebre, T.; Gebrehiwot, T.T.; Gebremedhin, A.T.; Geleijnse, J.M.; Gessner, B.D.; Gibney, K.B.; Ginawi, I.A.M.; Giref, A.Z.; Giroud, M.; Gishu, M.D.; Giussani, G.; Glaser, E.; Godwin, W.W.; Gomez-Dantes, H.; Gona, P.; Goodridge, A.; Gopalani, S.V.; Gotay, C.C.; Goto, A.; Gouda, H.N.; Grainger, R.; Greaves, F.; Guillemin, F.; Guo, Y.; Gupta, R.; Gupta, R.; Gupta, V.; Gutiérrez, R.A.; Haile, D.; Hailu, A.D.; Hailu, G.B.; Halasa, Y.A.; Hamadeh, R.R.; Hamidi, S.; Hammami, M.; Hancock, J.; Handal, A.J.; Hankey, G.J.; Hao, Y.; Harb, H.L.; Harikrishnan, S.; Haro, J.M.; Havmoeller, R.; Hay, R.J.; Heredia-Pi, I.B.; Heydarpour, P.; Hoek, H.W.; Horino, M.; Horita, N.; Hosgood, H.D.; Hoy, D.G.; Htet, A.S.; Huang, H.; Huang, J.J.; Huynh, C.; Iannarone, M.; Iburg, K.M.; Innos, K.; Inoue, M.; Iyer, V.J.; Jacobsen, K.H.; Jahanmehr, N.; Jakovljevic, M.B.; Javanbakht, M.; Jayaraman, S.P.; Jayatilleke, A.U.; Jee, S.H.; Jeemon, P.; Jensen, P.N.; Jiang, Y.; Jibat, T.; Jimenez-Corona, A.; Jin, Y.; Jonas, J.B.; Kabir, Z.; Kalkonde, Y.; Kamal, R.; Kan, H.; Karch, A.; Karema, C.K.; Karimkhani, C.; Kasaeian, A.; Kaul, A.; Kawakami, N.; Keiyoro, P.N.; Kemp, A.H.; Keren, A.; Kesavachandran, C.N.; Khader, Y.S.; Khan, A.R.; Khan, E.A.; Khang, Y-H.; Khera, S.; Khoja, T.A.M.; Khubchandani, J.; Kieling, C.; Kim, P.; Kim, C.; Kim, D.; Kim, Y.J.; Kissoon, N.; Knibbs, L.D.; Knudsen, A.K.; Kokubo, Y.; Kolte, D.; Kopec, J.A.; Kosen, S.; Kotsakis, G.A.; Koul, P.A.; Koyanagi, A.; Kravchenko, M.; Defo, B.K.; Bicer, B.K.; Kudom, A.A.; Kuipers, E.J.; Kumar, G.A.; Kutz, M.; Kwan, G.F.; Lal, A.; Lalloo, R.; Lallukka, T.; Lam, H.; Lam, J.O.; Langan, S.M.; Larsson, A.; Lavados, P.M.; Leasher, J.L.; Leigh, J.; Leung, R.; Levi, M.; Li, Y.; Li, Y.; Liang, J.; Liu, S.; Liu, Y.; Lloyd, B.K.; Lo, W.D.; Logroscino, G.; Looker, K.J.; Lotufo, P.A.; Lunevicius, R.; Lyons, R.A.; Mackay, M.T.; Magdy, M.; Razek, A.E.; Mahdavi, M.; Majdan, M.; Majeed, A.; Malekzadeh, R.; Marcenes, W.; Margolis, D.J.; Martinez-Raga, J.; Masiye, F.; Massano, J.; McGarvey, S.T.; McGrath, J.J.; McKee, M.; McMahon, B.J.; Meaney, P.A.; Mehari, A.; Mejia-Rodriguez, F.; Mekonnen, A.B.; Melaku, Y.A.; Memiah, P.; Memish, Z.A.; Mendoza, W.; Meretoja, A.; Meretoja, T.J.; Mhimbira, F.A.; Millear, A.; Miller, T.R.; Mills, E.J.; Mirarefin, M.; Mitchell, P.B.; Mock, C.N.; Mohammadi, A.; Mohammed, S.; Monasta, L.; Hernandez, J.C.M.; Montico, M.; Mooney, M.D.; Moradi-Lakeh, M.; Morawska, L.; Mueller, U.O.; Mullany, E.; Mumford, J.E.; Murdoch, M.E.; Nachega, J.B.; Nagel, G.; Naheed, A.; Naldi, L.; Nangia, V.; Newton, J.N.; Ng, M.; Ngalesoni, F.N.; Nguyen, Q.L.; Nisar, M.I.; Pete, P.M.N.; Nolla, J.M.; Norheim, O.F.; Norman, R.E.; Norrving, B.; Nunes, B.P.; Ogbo, F.A.; Oh, I-H.; Ohkubo, T.; Olivares, P.R.; Olusanya, B.O.; Olusanya, J.O.; Ortiz, A.; Osman, M.; Ota, E.; Pa, M.; Park, E-K.; Parsaeian, M.; de Azeredo, P.V.M.; Caicedo, A.J.P.; Patten, S.B.; Patton, G.C.; Pereira, D.M.; Perez-Padilla, R.; Perico, N.; Pesudovs, K.; Petzold, M.; Phillips, M.R.; Piel, F.B.; Pillay, J.D.; Pishgar, F.; Plass, D.; Platts-Mills, J.A.; Polinder, S.; Pond, C.D.; Popova, S.; Poulton, R.G.; Pourmalek, F.; Prabhakaran, D.; Prasad, N.M.; Qorbani, M.; Rabiee, R.H.S.; Radfar, A.; Rafay, A.; Rahimi, K.; Rahimi-Movaghar, V.; Rahman, M.; Rahman, M.H.U.; Rahman, S.U.; Rai, R.K.; Rajsic, S.; Ram, U.; Rao, P.; Refaat, A.H.; Reitsma, M.B.; Remuzzi, G.; Resnikoff, S.; Reynolds, A.; Ribeiro, A.L.; Blancas, M.J.R.; Roba, H.S.; Rojas-Rueda, D.; Ronfani, L.; Roshandel, G.; Roth, G.A.; Rothenbacher, D.; Roy, A.; Sagar, R.; Sahathevan, R.; Sanabria, J.R.; Sanchez-Niño, M.D.; Santos, I.S.; Santos, J.V.; Sarmiento-Suarez, R.; Sartorius, B.; Satpathy, M.; Savic, M.; Sawhney, M.; Schaub, M.P.; Schmidt, M.I.; Schneider, I.J.C.; Schöttker, B.; Schwebel, D.C.; Scott, J.G.; Seedat, S.; Sepanlou, S.G.; Servan-Mori, E.E.; Shackelford, K.A.; Shaheen, A.; Shaikh, M.A.; Sharma, R.; Sharma, U.; Shen, J.; Shepard, D.S.; Sheth, K.N.; Shibuya, K.; Shin, M-J.; Shiri, R.; Shiue, I.; Shrime, M.G.; Sigfusdottir, I.D.; Silva, D.A.S.; Silveira, D.G.A.; Singh, A.; Singh, J.A.; Singh, O.P.; Singh, P.K.; Sivonda, A.; Skirbekk, V.; Skogen, J.C.; Sligar, A.; Sliwa, K.; Soljak, M.; Søreide, K.; Sorensen, R.J.D.; Soriano, J.B.; Sposato, L.A.; Sreeramareddy, C.T.; Stathopoulou, V.; Steel, N.; Stein, D.J.; Steiner, T.J.; Steinke, S.; Stovner, L.; Stroumpoulis, K.; Sunguya, B.F.; Sur, P.; Swaminathan, S.; Sykes, B.L.; Szoeke, C.E.I.; Tabarés-Seisdedos, R.; Takala, J.S.; Tandon, N.; Tanne, D.; Tavakkoli, M.; Taye, B.; Taylor, H.R.; Ao, B.J.T.; Tedla, B.A.; Terkawi, A.S.; Thomson, A.J.; Thorne-Lyman, A.L.; Thrift, A.G.; Thurston, G.D.; Tobe-Gai, R.; Tonelli, M.; Topor-Madry, R.; Topouzis, F.; Tran, B.X.; Truelsen, T.; Dimbuene, Z.T.; Tsilimbaris, M.; Tura, A.K.; Tuzcu, E.M.; Tyrovolas, S.; Ukwaja, K.N.; Undurraga, E.A.; Uneke, C.J.; Uthman, O.A.; van Gool, C.H.; Varakin, Y.Y.; Vasankari, T.; Venketasubramanian, N.; Verma, R.K.; Violante, F.S.; Vladimirov, S.K.; Vlassov, V.V.; Vollset, S.E.; Wagner, G.R.; Waller, S.G.; Wang, L.; Watkins, D.A.; Weichenthal, S.; Weiderpass, E.; Weintraub, R.G.; Werdecker, A.; Westerman, R.; White, R.A.; Williams, H.C.; Wiysonge, C.S.; Wolfe, C.D.A.; Won, S.; Woodbrook, R.; Wubshet, M.; Xavier, D.; Xu, G.; Yadav, A.K.; Yan, L.L.; Yano, Y.; Yaseri, M.; Ye, P.; Yebyo, H.G.; Yip, P.; Yonemoto, N.; Yoon, S-J.; Younis, M.Z.; Yu, C.; Zaidi, Z.; Zaki, M.E.S.; Zeeb, H.; Zhou, M.; Zodpey, S.; Zuhlke, L.J.; Murray, C.J.L. Global, regional, and national incidence, prevalence, and years lived with disability for 310 diseases and injuries, 1990-2015: a systematic analysis for the Global Burden of Disease Study 2015.. Lancet.

[5] Santomauro D.F., Mantilla H.A.M., Shadid J., Zheng P., Ashbaugh C., Pigott D.M., Abbafati C., Adolph C., Amlag J.O., Aravkin A.Y., Bang-Jensen B.L., Bertolacci G.J., Bloom S.S., Castellano R., Castro E., Chakrabarti S., Chattopadhyay J., Cogen R.M., Collins J.K., Dai X., Dangel W.J., Dapper C., Deen A., Erickson M., Ewald S.B., Flaxman A.D., Frostad J.J., Fullman N., Giles J.R., Giref A.Z., Guo G., He J., Helak M., Hulland E.N., Idrisov B., Lindstrom A., Linebarger E., Lotufo P.A., Lozano R., Magistro B., Malta D.C., Månsson J.C., Marinho F., Mokdad A.H., Monasta L., Naik P., Nomura S., O’Halloran J.K., Ostroff S.M., Pasovic M., Penberthy L., Reiner R.C., Reinke G., Ribeiro A.L.P., Sholokhov A., Sorensen R.J.D., Varavikova E., Vo A.T., Walcott R., Watson S., Wiysonge C.S., Zigler B., Hay S.I., Vos T., Murray C.J.L., Whiteford H.A., Ferrari A.J. (2021). Global prevalence and burden of depressive and anxiety disorders in 204 countries and territories in 2020 due to the COVID-19 pandemic.. Lancet.

[6] https://developingchild.harvard.edu/science/key-concepts/toxic-stress/.

[7] Garner A.S., Shonkoff J.P., Siegel B.S., Dobbins M.I., Earls M.F., Garner A.S., McGuinn L., Pascoe J., Wood D.L. (2012). Committee on psychosocial aspects of child and family health; committee on early childhood, adoption, and dependent care; section on developmental and behavioral pediatrics. Early childhood adversity, toxic stress, and the role of the pediatrician: translating developmental science into lifelong health.. Pediatrics.

[8] Kessler R.C., Berglund P., Demler O., Jin R., Merikangas K.R., Walters E.E. (2005). Lifetime prevalence and age-of-onset distributions of DSM-IV disorders in the National Comorbidity Survey Replication.. Arch. Gen. Psychiatry.

[9] Smoller J.W. (2016). The genetics of stress-related disorders: PTSD, depression, and anxiety disorders.. Neuropsychopharmacology.

[10] Coleman J.R.I., Gaspar H.A., Bryois J., Breen G., Byrne E.M., Forstner A.J., Holmans P.A., de Leeuw C.A., Mattheisen M., McQuillin A., Whitehead Pavlides J.M., Pers T.H., Ripke S., Stahl E.A., Steinberg S., Trubetskoy V., Trzaskowski M., Wang Y., Abbott L., Abdellaoui A., Adams M.J., Adolfsson A.N., Agerbo E., Akil H., Albani D., Alliey-Rodriguez N., Als T.D., Andlauer T.F.M., Anjorin A., Antilla V., Van der Auwera S., Awasthi S., Bacanu S-A., Badner J.A., Bækvad-Hansen M., Barchas J.D., Bass N., Bauer M., Beekman A.T.F., Belliveau R., Bergen S.E., Bigdeli T.B., Binder E.B., Bøen E., Boks M., Boocock J., Budde M., Bunney W., Burmeister M., Buttenschøn H.N., Bybjerg-Grauholm J., Byerley W., Cai N., Casas M., Castelao E., Cerrato F., Cervantes P., Chambert K., Charney A.W., Chen D., Christensen J.H., Churchhouse C., St Clair D., Clarke T-K., Colodro-Conde L., Coryell W., Couvy-Duchesne B., Craig D.W., Crawford G.E., Cruceanu C., Czerski P.M., Dale A.M., Davies G., Deary I.J., Degenhardt F., Del-Favero J., DePaulo J.R., Derks E.M., Direk N., Djurovic S., Dobbyn A.L., Dolan C.V., Dumont A., Dunn E.C., Eley T.C., Elvsåshagen T., Escott-Price V., Fan C.C., Finucane H.K., Fischer S.B., Flickinger M., Foo J.C., Foroud T.M., Forty L., Frank J., Fraser C., Freimer N.B., Frisén L., Gade K., Gage D., Garnham J., Giambartolomei C., Goes F.S., Goldstein J., Gordon S.D., Gordon-Smith K., Green E.K., Green M.J., Greenwood T.A., Grove J., Guan W., Hall L.S., Hamshere M.L., Hansen C.S., Hansen T.F., Hautzinger M., Heilbronner U., van Hemert A.M., Herms S., Hickie I.B., Hipolito M., Hoffmann P., Holland D., Homuth G., Horn C., Hottenga J-J., Huckins L., Ising M., Jamain S., Jansen R., Johnson J.S., de Jong S., Jorgenson E., Juréus A., Kandaswamy R., Karlsson R., Kennedy J.L., Hassan Kiadeh F.F., Kittel-Schneider S., Knowles J.A., Kogevinas M., Kohane I.S., Koller A.C., Kraft J., Kretzschmar W.W., Krogh J., Kupka R., Kutalik Z., Lavebratt C., Lawrence J., Lawson W.B., Leber M., Lee P.H., Levy S.E., Li J.Z., Li Y., Lind P.A., Liu C., Olde Loohuis L.M., Maaser A., MacIntyre D.J., MacKinnon D.F., Mahon P.B., Maier W., Maier R.M., Marchini J., Martinsson L., Mbarek H., McCarroll S., McGrath P., McGuffin P., McInnis M.G., McKay J.D., Medeiros H., Medland S.E., Mehta D., Meng F., Middeldorp C.M., Mihailov E., Milaneschi Y., Milani L., Mirza S.S., Mondimore F.M., Montgomery G.W., Morris D.W., Mostafavi S., Mühleisen T.W., Mullins N., Nauck M., Ng B., Nguyen H., Nievergelt C.M., Nivard M.G., Nwulia E.A., Nyholt D.R., O’Donovan C., O’Reilly P.F., Ori A.P.S., Oruc L., Ösby U., Oskarsson H., Painter J.N., Parra J.G., Pedersen C.B., Pedersen M.G., Perry A., Peterson R.E., Pettersson E., Peyrot W.J., Pfennig A., Pistis G., Purcell S.M., Quiroz J.A., Qvist P., Regeer E.J., Reif A., Reinbold C.S., Rice J.P., Riley B.P., Rivas F., Rivera M., Roussos P., Ruderfer D.M., Ryu E., Sánchez-Mora C., Schatzberg A.F., Scheftner W.A., Schoevers R., Schork N.J., Schulte E.C., Shehktman T., Shen L., Shi J., Shilling P.D., Shyn S.I., Sigurdsson E., Slaney C., Smeland O.B., Smit J.H., Smith D.J., Sobell J.L., Spijker A.T., Steffens M., Strauss J.S., Streit F., Strohmaier J., Szelinger S., Tansey K.E., Teismann H., Teumer A., Thompson R.C., Thompson W., Thomson P.A., Thorgeirsson T.E., Traylor M., Treutlein J., Uitterlinden A.G., Umbricht D., Vedder H., Viktorin A., Visscher P.M., Wang W., Watson S.J., Webb B.T., Weickert C.S., Weickert T.W., Weinsheimer S.M., Wellmann J., Willemsen G., Witt S.H., Wu Y., Xi H.S., Xu W., Yang J., Young A.H., Zandi P., Zhang P., Zhang F., Zollner S., Adolfsson R., Agartz I., Alda M., Arolt V., Backlund L., Baune B.T., Bellivier F., Berger K., Berrettini W.H., Biernacka J.M., Blackwood D.H.R., Boehnke M., Boomsma D.I., Corvin A., Craddock N., Daly M.J., Dannlowski U., Domenici E., Domschke K., Esko T., Etain B., Frye M., Fullerton J.M., Gershon E.S., de Geus E.J.C., Gill M., Goes F., Grabe H.J., Grigoroiu-Serbanescu M., Hamilton S.P., Hauser J., Hayward C., Heath A.C., Hougaard D.M., Hultman C.M., Jones I., Jones L.A., Kahn R.S., Kendler K.S., Kirov G., Kloiber S., Landén M., Leboyer M., Lewis G., Li Q.S., Lissowska J., Lucae S., Madden P.A.F., Magnusson P.K., Martin N.G., Mayoral F., McElroy S.L., McIntosh A.M., McMahon F.J., Melle I., Metspalu A., Mitchell P.B., Morken G., Mors O., Mortensen P.B., Müller-Myhsok B., Myers R.M., Neale B.M., Nimgaonkar V., Nordentoft M., Nöthen M.M., O’Donovan M.C., Oedegaard K.J., Owen M.J., Paciga S.A., Pato C., Pato M.T., Pedersen N.L., Penninx B.W.J.H., Perlis R.H., Porteous D.J., Posthuma D., Potash J.B., Preisig M., Ramos-Quiroga J.A., Ribasés M., Rietschel M., Rouleau G.A., Schaefer C., Schalling M., Schofield P.R., Schulze T.G., Serretti A., Smoller J.W., Stefansson H., Stefansson K., Stordal E., Tiemeier H., Turecki G., Uher R., Vaaler A.E., Vieta E., Vincent J.B., Völzke H., Weissman M.M., Werge T., Andreassen O.A., Børglum A.D., Cichon S., Edenberg H.J., Di Florio A., Kelsoe J., Levinson D.F., Lewis C.M., Nurnberger J.I., Ophoff R.A., Scott L.J., Sklar P., Sullivan P.F., Wray N.R., Byrne E.M., Forstner A.J., Holmans P.A., de Leeuw C.A., Mattheisen M., McQuillin A., Whitehead P.J.M., Pers T.H., Ripke S., Stahl E.A., Steinberg S., Trubetskoy V., Trzaskowski M., Wang Y., Abbott L., Abdellaoui A., Adams M.J., Adolfsson A.N., Agerbo E., Akil H., Albani D., Alliey-Rodriguez N., Als T.D., Andlauer T.F.M., Anjorin A., Antilla V., Van der Auwera S., Awasthi S., Bacanu S-A., Badner J.A., Bækvad-Hansen M., Barchas J.D., Bass N., Bauer M., Beekman A.T.F., Belliveau R., Bergen S.E., Bigdeli T.B., Binder E.B., Bøen E., Boks M., Boocock J., Budde M., Bunney W., Burmeister M., Buttenschøn H.N., Bybjerg-Grauholm J., Byerley W., Cai N., Casas M., Castelao E., Cerrato F., Cervantes P., Chambert K., Charney A.W., Chen D., Christensen J.H., Churchhouse C., St Clair D., Clarke T-K., Colodro-Conde L., Coryell W., Couvy-Duchesne B., Craig D.W., Crawford G.E., Cruceanu C., Czerski P.M., Dale A.M., Davies G., Deary I.J., Degenhardt F., Del-Favero J., DePaulo J.R., Derks E.M., Direk N., Djurovic S., Dobbyn A.L., Dolan C.V., Dumont A., Dunn E.C., Eley T.C., Elvsåshagen T., Escott-Price V., Fan C.C., Finucane H.K., Fischer S.B., Flickinger M., Foo J.C., Foroud T.M., Forty L., Frank J., Fraser C., Freimer N.B., Frisén L., Gade K., Gage D., Garnham J., Giambartolomei C., Goes F.S., Goldstein J., Gordon S.D., Gordon-Smith K., Green E.K., Green M.J., Greenwood T.A., Grove J., Guan W., Hall L.S., Hamshere M.L., Hansen C.S., Hansen T.F., Hautzinger M., Heilbronner U., van Hemert A.M., Herms S., Hickie I.B., Hipolito M., Hoffmann P., Holland D., Homuth G., Horn C., Hottenga J-J., Huckins L., Ising M., Jamain S., Jansen R., Johnson J.S., de Jong S., Jorgenson E., Juréus A., Kandaswamy R., Karlsson R., Kennedy J.L., Hassan Kiadeh F.F., Kittel-Schneider S., Knowles J.A., Kogevinas M., Kohane I.S., Koller A.C., Kraft J., Kretzschmar W.W., Krogh J., Kupka R., Kutalik Z., Lavebratt C., Lawrence J., Lawson W.B., Leber M., Lee P.H., Levy S.E., Li J.Z., Li Y., Lind P.A., Liu C., Olde Loohuis L.M., Maaser A., MacIntyre D.J., MacKinnon D.F., Mahon P.B., Maier W., Maier R.M., Marchini J., Martinsson L., Mbarek H., McCarroll S., McGrath P., McGuffin P., McInnis M.G., McKay J.D., Medeiros H., Medland S.E., Mehta D., Meng F., Middeldorp C.M., Mihailov E., Milaneschi Y., Milani L., Mirza S.S., Mondimore F.M., Montgomery G.W., Morris D.W., Mostafavi S., Mühleisen T.W., Mullins N., Nauck M., Ng B., Nguyen H., Nievergelt C.M., Nivard M.G., Nwulia E.A., Nyholt D.R., O’Donovan C., O’Reilly P.F., Ori A.P.S., Oruc L., Ösby U., Oskarsson H., Painter J.N., Parra J.G., Pedersen C.B., Pedersen M.G., Perry A., Peterson R.E., Pettersson E., Peyrot W.J., Pfennig A., Pistis G., Purcell S.M., Quiroz J.A., Qvist P., Regeer E.J., Reif A., Reinbold C.S., Rice J.P., Riley B.P., Rivas F., Rivera M., Roussos P., Ruderfer D.M., Ryu E., Sánchez-Mora C., Schatzberg A.F., Scheftner W.A., Schoevers R., Schork N.J., Schulte E.C., Shehktman T., Shen L., Shi J., Shilling P.D., Shyn S.I., Sigurdsson E., Slaney C., Smeland O.B., Smit J.H., Smith D.J., Sobell J.L., Spijker A.T., Steffens M., Strauss J.S., Streit F., Strohmaier J., Szelinger S., Tansey K.E., Teismann H., Teumer A., Thompson R.C., Thompson W., Thomson P.A., Thorgeirsson T.E., Traylor M., Treutlein J., Uitterlinden A.G., Umbricht D., Vedder H., Viktorin A., Visscher P.M., Wang W., Watson S.J., Webb B.T., Weickert C.S., Weickert T.W., Weinsheimer S.M., Wellmann J., Willemsen G., Witt S.H., Wu Y., Xi H.S., Xu W., Yang J., Young A.H., Zandi P., Zhang P., Zhang F., Zollner S., Adolfsson R., Agartz I., Alda M., Arolt V., Backlund L., Baune B.T., Bellivier F., Berger K., Berrettini W.H., Biernacka J.M., Blackwood D.H.R., Boehnke M., Boomsma D.I., Corvin A., Craddock N., Daly M.J., Dannlowski U., Domenici E., Domschke K., Esko T., Etain B., Frye M., Fullerton J.M., Gershon E.S., de Geus E.J.C., Gill M., Goes F., Grabe H.J., Grigoroiu-Serbanescu M., Hamilton S.P., Hauser J., Hayward C., Heath A.C., Hougaard D.M., Hultman C.M., Jones I., Jones L.A., Kahn R.S., Kendler K.S., Kirov G., Kloiber S., Landén M., Leboyer M., Lewis G., Li Q.S., Lissowska J., Lucae S., Madden P.A.F., Magnusson P.K., Martin N.G., Mayoral F., McElroy S.L., McIntosh A.M., McMahon F.J., Melle I., Metspalu A., Mitchell P.B., Morken G., Mors O., Mortensen P.B., Müller-Myhsok B., Myers R.M., Neale B.M., Nimgaonkar V., Nordentoft M., Nöthen M.M., O’Donovan M.C., Oedegaard K.J., Owen M.J., Paciga S.A., Pato C., Pato M.T., Pedersen N.L., Penninx B.W.J.H., Perlis R.H., Porteous D.J., Posthuma D., Potash J.B., Preisig M., Ramos-Quiroga J.A., Ribasés M., Rietschel M., Rouleau G.A., Schaefer C., Schalling M., Schofield P.R., Schulze T.G., Serretti A., Smoller J.W., Stefansson H., Stefansson K., Stordal E., Tiemeier H., Turecki G., Uher R., Vaaler A.E., Vieta E., Vincent J.B., Völzke H., Weissman M.M., Werge T., Andreassen O.A., Børglum A.D., Cichon S., Edenberg H.J., Di Florio A., Kelsoe J., Levinson D.F., Lewis C.M., Nurnberger J.I., Ophoff R.A., Scott L.J., Sklar P., Sullivan P.F., Wray N.R. (2020). The Genetics of the Mood Disorder Spectrum: Genome-wide Association Analyses of More Than 185,000 Cases and 439,000 Controls.. Biol. Psychiatry.

[11] Kendler K.S., Gatz M., Gardner C.O., Pedersen N.L. (2007). Clinical indices of familial depression in the Swedish Twin Registry.. Acta Psychiatr. Scand..

[12] Hettema J.M., Kettenmann B., Ahluwalia V., McCarthy C., Kates W.R., Schmitt J.E., Silberg J.L., Neale M.C., Kendler K.S., Fatouros P. (2012). Pilot multimodal twin imaging study of generalized anxiety disorder.. Depress. Anxiety.

[13] Berardis D., Marini S., Serroni N., Iasevoli F., Tomasetti C., Bartolomeis A., Mazza M., Tempesta D., Valchera A., Fornaro M., Pompili M., Sepede G., Vellante F., Orsolini L., Martinotti G., Giannantonio M. (2015). Targeting the noradrenergic system in posttraumatic stress disorder: A systematic review and meta-analysis of prazosin trials.. Curr. Drug Targets.

[14] Tempesta D., Mazza M., Serroni N., Moschetta F.S., Di Giannantonio M., Ferrara M., De Berardis D. (2013). Neuropsychological functioning in young subjects with generalized anxiety disorder with and without pharmacotherapy.. Prog. Neuropsychopharmacol. Biol. Psychiatry.

[15] Ventriglio A., Bhugra D., Sampogna G., Luciano M., De Berardis D., Sani G., Fiorillo A. (2020). From dysthymia to treatment-resistant depression: Evolution of a psychopathological construct.. Int. Rev. Psychiatry.

[16] Michopoulos V., Powers A., Gillespie C.F., Ressler K.J., Jovanovic T. (2017). Inflammation in fear- and anxiety-based disorders: PTSD, GAD, and beyond.. Neuropsychopharmacology.

[17] Serra-Blasco M., Radua J., Soriano-Mas C., Gómez-Benlloch A., Porta-Casteràs D., Carulla-Roig M., Albajes-Eizagirre A., Arnone D., Klauser P., Canales-Rodríguez E.J., Hilbert K., Wise T., Cheng Y., Kandilarova S., Mataix-Cols D., Vieta E., Via E., Cardoner N. (2021). Structural brain correlates in major depression, anxiety disorders and post-traumatic stress disorder: A voxel-based morphometry meta-analysis.. Neurosci. Biobehav. Rev..

[18] Verbitsky A., Dopfel D., Zhang N. (2020). Rodent models of post-traumatic stress disorder: Behavioral assessment.. Transl. Psychiatry.

[19] Planchez B., Surget A., Belzung C. (2019). Animal models of major depression: Drawbacks and challenges.. J. Neural Transm..

[20] Yehuda R., Antelman S.M. (1993). Criteria for rationally evaluating animal models of postraumatic stress disorder.. Biol. Psychiatry.

[21] Franklin T.B., Saab B.J., Mansuy I.M. (2012). Neural mechanisms of stress resilience and vulnerability.. Neuron.

[22] Armario A., Escorihuela R.M., Nadal R. (2008). Long-term neuroendocrine and behavioural effects of a single exposure to stress in adult animals.. Neurosci. Biobehav. Rev..

[23] Campos A.C., Fogaça M.V., Aguiar D.C., Guimarães F.S. (2013). Animal models of anxiety disorders and stress.. Rev. Bras. Psiquiatr..

[24] Finnell J.E., Lombard C.M., Padi A.R., Moffitt C.M., Wilson L.B., Wood C.S., Wood S.K. (2017). Physical versus psychological social stress in male rats reveals distinct cardiovascular, inflammatory and behavioral consequences.. PLoS One.

[25] Márquez C., Belda X., Armario A. (2002). Post-stress recovery of pituitary-adrenal hormones and glucose, but not the response during exposure to the stressor, is a marker of stress intensity in highly stressful situations.. Brain Res..

[26] Belda X., Fuentes S., Nadal R., Armario A. (2008). A single exposure to immobilization causes long-lasting pituitary-adrenal and behavioral sensitization to mild stressors.. Horm. Behav..

[27] Andero R., Heldt S.A., Ye K., Liu X., Armario A., Ressler K.J. (2011). Effect of 7,8-dihydroxyflavone, a small-molecule TrkB agonist, on emotional learning.. Am. J. Psychiatry.

[28] Velasco E.R., Florido A., Flores Á., Senabre E., Gomez-Gomez A., Torres A., Roca A., Norrholm S., Newman E.L., Das P., Ross R.A., Lori A., Pozo O.J., Ressler K.J., Garcia-Esteve L.L., Jovanovic T., Andero R. (2022). PACAP-PAC1R modulates fear extinction via the ventromedial hypothalamus.. Nat. Commun..

[29] Sanz-García A., Knafo S., Pereda-Pérez I., Esteban J.A., Venero C., Armario A. (2016). Administration of the TrkB receptor agonist 7,8-dihydroxyflavone prevents traumatic stress-induced spatial memory deficits and changes in synaptic plasticity.. Hippocampus.

[30] Zhang J.H., Han F., Shi Y.X. (2012). Single prolonged stress induces changes in the expression of mineralocorticoid receptor in the medial prefrontal cortex in a rat model of post-traumatic stress disorder.. Mol. Med. Rep..

[31] Willner P. (1984). The validity of animal models of depression.. Psychopharmacology.

[32] Willner P., Belzung C. (2015). Treatment-resistant depression: are animal models of depression fit for purpose?. Psychopharmacology.

[33] Willner P., Scheel-Krüger J., Belzung C. (2013). The neurobiology of depression and antidepressant action.. Neurosci. Biobehav. Rev..

[34] Belzung C., Willner P., Philippot P. (2015). Depression: From psychopathology to pathophysiology.. Curr. Opin. Neurobiol..

[35] Badcock P., Allen N. (2003). Adaptive social reasoning in depressed mood and depressive vulnerability.. Cogn. Emotion.

[36] Berton O., McClung C.A., DiLeone R.J., Krishnan V., Renthal W., Russo S.J. (2006). Essential role of BDNF in the mesolimbic dopamine pathway in social defeat stress.. Science.

[37] Björkqvist K. (2001). Social defeat as a stressor in humans.. Physiol. Behav..

[38] Rohde P. (2001). The relevance of hierarchies, territories, defeat for depression in humans: Hypotheses and clinical predictions.. J. Affect. Disord..

[39] Hultman R., Mague S.D., Li Q., Katz B.M., Michel N., Lin L., Wang J., David L.K., Blount C., Chandy R., Carlson D., Ulrich K., Carin L., Dunson D., Kumar S., Deisseroth K., Moore S.D., Dzirasa K. (2016). Dysregulation of prefrontal cortex-mediated slow-evolving limbic dynamics drives stress-induced emotional pathology.. Neuron.

[40] Han Q.Q., Yang L., Huang H.J., Wang Y.L., Yu R., Wang J., Pilot A., Wu G.C., Liu Q., Yu J. (2017). Differential GR expression and translocation in the hippocampus mediates susceptibility vs. resilience to chronic social defeat stress.. Front. Neurosci..

[41] Tsankova N.M., Berton O., Renthal W., Kumar A., Neve R.L., Nestler E.J. (2006). Sustained hippocampal chromatin regulation in a mouse model of depression and antidepressant action.. Nat. Neurosci..

[42] Hill M.N., Hellemans K.G.C., Verma P., Gorzalka B.B., Weinberg J. (2012). Neurobiology of chronic mild stress: Parallels to major depression.. Neurosci. Biobehav. Rev..

[43] Willner P. (2017). The chronic mild stress (CMS) model of depression: History, evaluation and usage.. Neurobiol. Stress.

[44] Kalueff A.V., Wheaton M., Murphy D.L. (2007). What’s wrong with my mouse model?. Behav. Brain Res..

[45] Gale G.D., Anagnostaras S.G., Godsil B.P., Mitchell S., Nozawa T., Sage J.R., Wiltgen B., Fanselow M.S. (2004). Role of the basolateral amygdala in the storage of fear memories across the adult lifetime of rats.. J. Neurosci..

[46] McEwen B.S., De Kloet E.R., Rostene W. (1986). Adrenal steroid receptors and actions in the nervous system.. Physiol. Rev..

[47] Amaya J.M., Suidgeest E., Sahut-Barnola I., Dumontet T., Montanier N., Pagès G., Keller C., van der Weerd L., Pereira A.M., Martinez A., Meijer O.C. (2021). Effects of long-term endogenous corticosteroid exposure on brain volume and glial cells in the AdKO mouse.. Front. Neurosci..

[48] Wellman C.L. (2001). Dendritic reorganization in pyramidal neurons in medial prefrontal cortex after chronic corticosterone administration.. J. Neurobiol..

[49] Cook S.C., Wellman C.L. (2004). Chronic stress alters dendritic morphology in rat medial prefrontal cortex.. J. Neurobiol..

[50] Sapolsky R.M., Krey L.C., McEwen B.S. (1985). Prolonged glucocorticoid exposure reduces hippocampal neuron number: Implications for aging.. J. Neurosci..

[51] Sapolsky R.M., Uno H., Rebert C.S., Finch C.E. (1990). Hippocampal damage associated with prolonged glucocorticoid exposure in primates.. J. Neurosci..

[52] Mitra R., Sapolsky R.M. (2008). Acute corticosterone treatment is sufficient to induce anxiety and amygdaloid dendritic hypertrophy.. Proc. Natl. Acad. Sci. USA.

[53] Magarinos A.M., McEwen B.S. (1995). Stress-induced atrophy of apical dendrites of hippocampal CA3c neurons: Involvement of glucocorticoid secretion and excitatory amino acid receptors.. Neuroscience.

[54] Herman J.P., Patel P.D., Akil H., Watson S.J. (1989). Localization and regulation of glucocorticoid and mineralocorticoid receptor messenger RNAs in the hippocampal formation of the rat.. Mol. Endocrinol..

[55] Jacobson L., Sapolsky R. (1991). The role of the hippocampus in feedback regulation of the hypothalamic-pituitary-adrenocortical axis.. Endocr. Rev..

[56] Sapolsky R.M., Zola-Morgan S., Squire L.R. (1991). Inhibition of glucocorticoid secretion by the hippocampal formation in the primate.. J. Neurosci..

[57] Herman J.P., Cullinan W.E. (1997). Neurocircuitry of stress: central control of the hypothalamo-pituitary-adrenocortical axis.. Trends Neurosci..

[58] Alexandra K.M., Fenster R.J., Laurent E.S., Ressler K.J., Phelps E.A. (2022). Prefrontal cortex, amygdala, and threat processing: implications for PTSD.. Neuropsychopharmacology.

[59] Watanabe Y., Gould E., McEwen B.S. (1992). Stress induces atrophy of apical dendrites of hippocampal CA3 pyramidal neurons.. Brain Res..

[60] Magariños A.M., McEwen B.S., Flügge G., Fuchs E. (1996). Chronic psychosocial stress causes apical dendritic atrophy of hippocampal CA3 pyramidal neurons in subordinate tree shrews.. J. Neurosci..

[61] Conrad C.D., Galea L.A.M., Kuroda Y., McEwen B.S. (1996). Chronic stress impairs rat spatial memory on the Y maze, and this effect is blocked by tianeptine treatment.. Behav. Neurosci..

[62] Sousa N., Lukoyanov N.V., Madeira M.D., Almeida O.F.X., Paula-Barbosa M.M. (2000). Reorganization of the morphology of hippocampal neurites and synapses after stress-induced damage correlates with behavioral improvement.. Neuroscience.

[63] Kole M.H.P., Czéh B., Fuchs E. (2004). Homeostatic maintenance in excitability of tree shrew hippocampal CA3 pyramidal neurons after chronic stress.. Hippocampus.

[64] Vyas A., Mitra R., Shankaranarayana R.B.S., Chattarji S. (2002). Chronic stress induces contrasting patterns of dendritic remodeling in hippocampal and amygdaloid neurons.. J. Neurosci..

[65] Oitzl M.S., Champagne D.L., van der Veen R., de Kloet E.R. (2010). Brain development under stress: Hypotheses of glucocorticoid actions revisited.. Neurosci. Biobehav. Rev..

[66] Sapolsky R.M., Krey L.C., Mcewen B.S. (1986). The neuroendocrinology of stress and aging: The glucocorticoid cascade hypothesis.. Endocr. Rev..

[67] Conrad C.D. (2008). Chronic stress-induced hippocampal vulnerability: The glucocorticoid vulnerability hypothesis.. Rev. Neurosci..

[68] Andero R., Choi D.C., Ressler K.J. (2014). BDNF-TrkB receptor regulation of distributed adult neural plasticity, memory formation, and psychiatric disorders.. Prog. Mol. Biol. Transl. Sci..

[69] Duric V., Duman R.S. (2013). Depression and treatment response: Dynamic interplay of signaling pathways and altered neural processes.. Cell. Mol. Life Sci..

[70] Felger J.C., Lotrich F.E. (2013). Inflammatory cytokines in depression: Neurobiological mechanisms and therapeutic implications.. Neuroscience.

[71] Stepanichev M., Dygalo N.N., Grigoryan G., Shishkina G.T., Gulyaeva N. (2014). Rodent models of depression: Neurotrophic and neuroinflammatory biomarkers.. BioMed Res. Int..

[72] Lowy M.T., Gault L., Yamamoto B.K. (1993). Adrenalectomy attenuates stress-induced elevations in extracellular glutamate concentrations in the hippocampus.. J. Neurochem..

[73] Lowy M.T., Wittenberg L., Yamamoto B.K. (1995). Effect of acute stress on hippocampal glutamate levels and spectrin proteolysis in young and aged rats.. J. Neurochem..

[74] Reznikov L.R., Grillo C.A., Piroli G.G., Pasumarthi R.K., Reagan L.P., Fadel J. (2007). Acute stress-mediated increases in extracellular glutamate levels in the rat amygdala: differential effects of antidepressant treatment.. Eur. J. Neurosci..

[75] Venero C., Borrell J. (1999). Rapid glucocorticoid effects on excitatory amino acid levels in the hippocampus: a microdialysis study in freely moving rats.. Eur. J. Neurosci..

[76] Bagley J., Moghaddam B. (1997). Temporal dynamics of glutamate efflux in the prefrontal cortex and in the hippocampus following repeated stress: effects of pretreatment with saline or diazepam.. Neuroscience.

[77] Moghaddam B. (1993). Stress preferentially increases extraneuronal levels of excitatory amino acids in the prefrontal cortex: comparison to hippocampus and basal ganglia.. J. Neurochem..

[78] Martin K.P., Wellman C.L. (2011). NMDA receptor blockade alters stress-induced dendritic remodeling in medial prefrontal cortex.. Cereb. Cortex.

[79] Yin Y., Edelman G.M., Vanderklish P.W. (2002). The brain-derived neurotrophic factor enhances synthesis of Arc in synaptoneurosomes.. Proc. Natl. Acad. Sci. USA.

[80] Okuno H. (2011). Regulation and function of immediate-early genes in the brain: Beyond neuronal activity markers.. Neurosci. Res..

[81] Hunter C.J., Remenyi J., Correa S.A., Privitera L., Reyskens K.M.S.E., Martin K.J., Toth R., Frenguelli B.G., Arthur J.S.C. (2017). MSK1 regulates transcriptional induction of Arc/Arg3.1 in response to neurotrophins.. FEBS Open Bio.

[82] Robinson S., Mogul A.S., Taylor-Yeremeeva E.M., Khan A., Tirabassi A.D., Wang H.Y. (2021). Stress diminishes BDNF-stimulated TrkB signaling, TrkB-NMDA receptor linkage and neuronal activity in the rat brain.. Neuroscience.

[83] Messaoudi E., Ying S.W., Kanhema T., Croll S.D., Bramham C.R. (2002). Brain-derived neurotrophic factor triggers transcription-dependent, late phase long-term potentiation in vivo.. J. Neurosci..

[84] Ying S.W., Futter M., Rosenblum K., Webber M.J., Hunt S.P., Bliss T.V.P., Bramham C.R. (2002). Brain-derived neurotrophic factor induces long-term potentiation in intact adult hippocampus: requirement for ERK activation coupled to CREB and upregulation of Arc synthesis.. J. Neurosci..

[85] Bramham C.R., Alme M.N., Bittins M., Kuipers S.D., Nair R.R., Pai B., Panja D., Schubert M., Soule J., Tiron A., Wibrand K. (2010). The Arc of synaptic memory.. Exp. Brain Res..

[86] Bekinschtein P., Cammarota M., Katche C., Slipczuk L., Rossato J.I., Goldin A., Izquierdo I., Medina J.H. (2008). BDNF is essential to promote persistence of long-term memory storage.. Proc. Natl. Acad. Sci. USA.

[87] Bramham C.R., Messaoudi E. (2005). BDNF function in adult synaptic plasticity: The synaptic consolidation hypothesis.. Prog. Neurobiol..

[88] Schmidt H.D., Duman R.S. (2010). Peripheral BDNF produces antidepressant-like effects in cellular and behavioral models.. Neuropsychopharmacol..

[89] Zhang W., Wu J., Ward M.D., Yang S., Chuang Y.A., Xiao M., Li R., Leahy D.J., Worley P.F. (2015). Structural basis of arc binding to synaptic proteins: implications for cognitive disease.. Neuron.

[90] Dwivedi Y., Rizavi H.S., Conley R.R., Roberts R.C., Tamminga C.A., Pandey G.N. (2003). Altered gene expression of brain-derived neurotrophic factor and receptor tyrosine kinase B in postmortem brain of suicide subjects.. Arch. Gen. Psychiatry.

[91] Karege F., Vaudan G., Schwald M., Perroud N., La Harpe R. (2005). Neurotrophin levels in postmortem brains of suicide victims and the effects of antemortem diagnosis and psychotropic drugs.. Brain Res. Mol. Brain Res..

[92] Thompson R.M. (2011). Decreased BDNF, trkB-TK+ and GAD67 mRNA expression in the hippocampus of individuals with schizophrenia and mood disorders.. J. Psychiatry Neurosci..

[93] Nibuya M., Takahashi M., Russell D.S., Duman R.S. (1999). Repeated stress increases catalytic TrkB mRNA in rat hippocampus.. Neurosci. Lett..

[94] Kozlovsky N., Matar M.A., Kaplan Z., Kotler M., Zohar J., Cohen H. (2007). Long-term down-regulation of BDNF mRNA in rat hippocampal CA1 subregion correlates with PTSD-like behavioural stress response.. Int. J. Neuropsychopharmacol..

[95] Shi S.S., Shao S., Yuan B., Pan F., Li Z.L. (2010). Acute stress and chronic stress change brain-derived neurotrophic factor (BDNF) and tyrosine kinase-coupled receptor (TrkB) expression in both young and aged rat hippocampus.. Yonsei Med. J..

[96] Nasrallah P., Haidar E.A., Stephan J.S., El Hayek L., Karnib N., Khalifeh M., Barmo N., Jabre V., Houbeika R., Ghanem A., Nasser J., Zeeni N., Bassil M., Sleiman S.F. (2019). Branched-chain amino acids mediate resilience to chronic social defeat stress by activating BDNF/TRKB signaling.. Neurobiol. Stress.

[97] Dantzer R., O’Connor J.C., Freund G.G., Johnson R.W., Kelley K.W. (2008). From inflammation to sickness and depression: when the immune system subjugates the brain.. Nat. Rev. Neurosci..

[98] Koo J.W., Duman R.S. (2008). IL-1β is an essential mediator of the antineurogenic and anhedonic effects of stress.. Proc. Natl. Acad. Sci. USA.

[99] Miller A.H., Maletic V., Raison C.L. (2009). Inflammation and its discontents: The role of cytokines in the pathophysiology of major depression.. Biol. Psychiatry.

[100] Koo J.W., Russo S.J., Ferguson D., Nestler E.J., Duman R.S. (2010). Nuclear factor-κB is a critical mediator of stress-impaired neurogenesis and depressive behavior.. Proc. Natl. Acad. Sci. USA.

[101] Troubat R., Barone P., Leman S., Desmidt T., Cressant A., Atanasova B., Brizard B., El Hage W., Surget A., Belzung C., Camus V. (2021). Neuroinflammation and depression: A review.. Eur. J. Neurosci..

[102] Miller A.H., Haroon E., Raison C.L., Felger J.C. (2013). Cytokine targets in the brain: impact on neurotransmitters and neurocircuits.. Depress. Anxiety.

[103] Barrientos R.M., Sprunger D.B., Campeau S., Higgins E.A., Watkins L.R., Rudy J.W., Maier S.F. (2003). Brain-derived neurotrophic factor mRNA downregulation produced by social isolation is blocked by intrahippocampal interleukin-1 receptor antagonist.. Neuroscience.

[104] (2008). ben Menachem-Zidon, O.; Goshen, I.; Kreisel, T.; ben Menahem, Y.; Reinhartz, E.; ben Hur, T. Intrahippocampal transplantation of transgenic neural precursor cells overexpressing Interleukin-1 receptor antagonist blocks chronic isolation-induced impairment in memory and neurogenesis.. Neuropsychopharmacology.

[105] Wu C.W., Chen Y.C., Yu L., Chen H., Jen C.J., Huang A.M., Tsai H.J., Chang Y.T., Kuo Y.M. (2007). Treadmill exercise counteracts the suppressive effects of peripheral lipopolysaccharide on hippocampal neurogenesis and learning and memory.. J. Neurochem..

[106] Ida T., Hara M., Nakamura Y., Kozaki S., Tsunoda S., Ihara H. (2008). Cytokine-induced enhancement of calcium-dependent glutamate release from astrocytes mediated by nitric oxide.. Neurosci. Lett..

[107] Haydon P.G., Carmignoto G. (2006). Astrocyte control of synaptic transmission and neurovascular coupling.. Physiol. Rev..

[108] Gavillet M., Allaman I., Magistretti P.J. (2008). Modulation of astrocytic metabolic phenotype by proinflammatory cytokines.. Glia.

[109] Thornton P., Pinteaux E., Gibson R.M., Allan S.M., Rothwell N.J. (2006). Interleukin-1-induced neurotoxicity is mediated by glia and requires caspase activation and free radical release.. J. Neurochem..

[110] Pepys M.B., Hirschfield G.M. (2003). C-reactive protein: A critical update.. J. Clin. Invest..

[111] Köhler-Forsberg O., Buttenschøn H.N., Tansey K.E., Maier W., Hauser J., Dernovsek M.Z., Henigsberg N., Souery D., Farmer A., Rietschel M., McGuffin P., Aitchison K.J., Uher R., Mors O. (2017). Association between C-reactive protein (CRP) with depression symptom severity and specific depressive symptoms in major depression.. Brain Behav. Immun..

[112] De Berardis D., Campanella D., Gambi F., La Rovere R., Carano A., Conti C.M., Silvestrini C., Serroni N., Piersanti D., Di Giuseppe B., Moschetta F.S., Cotellessa C., Fulcheri M., Salerno R.M., Ferro F.M. (2006). The role of C-reactive protein in mood disorders.. Int. J. Immunopathol. Pharmacol..

[113] Hsuchou H., Kastin A.J., Pan W. (2012). Blood-borne metabolic factors in obesity exacerbate injury-induced gliosis.. J. Mol. Neurosci..

[114] Blossom V., Gokul M., Kumar N.A., Kini R.D., Nayak S., Bhagyalakshmi K. (2020). Chronic unpredictable stress-induced inflammation and quantitative analysis of neurons of distinct brain regions in Wistar rat model of comorbid depression.. Vet. World.

[115] Hamon M., Blier P. (2013). Monoamine neurocircuitry in depression and strategies for new treatments.. Prog. Neuropsychopharmacol. Biol. Psychiatry.

[116] Maddison D.C., Giorgini F. (2015). The kynurenine pathway and neurodegenerative disease.. Semin. Cell Dev. Biol..

[117] Henckens M.J.A.G., van der Marel K., van der Toorn A., Pillai A.G., Fernández G., Dijkhuizen R.M., Joëls M. (2015). Stress-induced alterations in large-scale functional networks of the rodent brain.. Neuroimage.

[118] Anacker C., Scholz J., O’Donnell K.J., Allemang-Grand R., Diorio J., Bagot R.C., Nestler E.J., Hen R., Lerch J.P., Meaney M.J. (2016). Neuroanatomic differences associated with stress susceptibility and resilience.. Biol. Psychiatry.

[119] Magalhães R., Bourgin J., Boumezbeur F., Marques P., Bottlaender M., Poupon C., Djemaï B., Duchesnay E., Mériaux S., Sousa N., Jay T.M., Cachia A. (2017). White matter changes in microstructure associated with a maladaptive response to stress in rats.. Transl. Psychiatry.

[120] Magalhães R., Barrière D.A., Novais A., Marques F., Marques P., Cerqueira J., Sousa J.C., Cachia A., Boumezbeur F., Bottlaender M., Jay T.M., Mériaux S., Sousa N. (2018). The dynamics of stress: A longitudinal MRI study of rat brain structure and connectome.. Mol. Psychiatry.

[121] Liu X., Yuan J., Guang Y., Wang X., Feng Z. (2018). Longitudinal in vivo diffusion tensor imaging detects differential microstructural alterations in the hippocampus of chronic social defeat stress-susceptible and resilient mice.. Front. Neurosci..

[122] Nagy S.A., Vranesics A., Varga Z., Csabai D., Bruszt N., Bali Z.K., Perlaki G., Hernádi I., Berente Z., Miseta A., Dóczi T., Czéh B. (2020). Stress-induced microstructural alterations correlate with the cognitive performance of rats: A longitudinal in vivo diffusion tensor imaging study.. Front. Neurosci..

[123] Luby J.L., Barch D., Whalen D., Tillman R., Belden A. (2017). Association between early life adversity and risk for poor emotional and physical health in adolescence a putative mechanistic neurodevelopmental pathway.. JAMA Pediatr..

[124] Sowell E.R., Peterson B.S., Thompson P.M., Welcome S.E., Henkenius A.L., Toga A.W. (2003). Mapping cortical change across the human life span.. Nat. Neurosci..

[125] Sturman D.A., Moghaddam B. (2011). The neurobiology of adolescence: Changes in brain architecture, functional dynamics, and behavioral tendencies.. Neurosci. Biobehav. Rev..

[126] Tau G.Z., Peterson B.S. (2010). Normal development of brain circuits.. Neuropsychopharmacology.

[127] Fox S.E., Levitt P., Nelson C.A. (2010). How the timing and quality of early experiences influence the development of brain architecture.. Child Dev..

[128] Gogtay N., Giedd J.N., Lusk L., Hayashi K.M., Greenstein D., Vaituzis A.C., Nugent T.F., Herman D.H., Clasen L.S., Toga A.W., Rapoport J.L., Thompson P.M. (2004). Dynamic mapping of human cortical development during childhood through early adulthood.. Proc. Natl. Acad. Sci. USA.

[129] Knickmeyer R.C., Gouttard S., Kang C., Evans D., Wilber K., Smith J.K., Hamer R.M., Lin W., Gerig G., Gilmore J.H. (2008). A structural MRI study of human brain development from birth to 2 years.. J. Neurosci..

[130] Kaczkurkin A.N., Raznahan A., Satterthwaite T.D. (2019). Sex differences in the developing brain: Insights from multimodal neuroimaging.. Neuropsychopharmacology.

[131] Hart H., Rubia K. (2012). Neuroimaging of child abuse: A critical review.. Front. Hum. Neurosci..

[132] Lupien S.J., McEwen B.S., Gunnar M.R., Heim C. (2009). Effects of stress throughout the lifespan on the brain, behaviour and cognition.. Nat. Rev. Neurosci..

[133] Bennett M., Lagopoulos J. (2019). Stress, trauma and synaptic plasticity..

[134] McEwen B.S., Nasca C., Gray J.D. (2016). Stress effects on neuronal structure: Hippocampus, amygdala, and prefrontal cortex.. Neuropsychopharmacology.

[135] Graham A.M., Doyle O., Tilden E.L., Sullivan E.L., Gustafsson H.C., Marr M., Allen M., Mackiewicz S.K.L. (2022). Effects of maternal psychological stress during pregnancy on offspring brain development: Considering the role of inflammation and potential for preventive intervention.. Biol. Psychiatry Cogn. Neurosci. Neuroimaging.

[136] De Asis-Cruz J., Andescavage N., Limperopoulos C. (2022). Adverse prenatal exposures and fetal brain development: Insights from advanced fetal magnetic resonance imaging.. Biol. Psychiatry Cogn. Neurosci. Neuroimaging.

[137] (2021). Lubczyńska, M.J.; Muetzel, R.L.; El Marroun, H.; Hoek, G.; Kooter, I.M.; Thomson, E.M.; Hillegers, M.; Vernooij, M.W.; White, T.; Tiemeier, H.; Guxens, M. Air pollution exposure during pregnancy and childhood and brain morphology in preadolescents.. Environ. Res..

[138] Triplett R.L., Lean R.E., Parikh A., Miller J.P., Alexopoulos D., Kaplan S., Meyer D., Adamson C., Smyser T.A., Rogers C.E., Barch D.M., Warner B., Luby J.L., Smyser C.D. (2022). Association of prenatal exposure to early-life adversity with neonatal brain volumes at birth.. JAMA Netw. Open.

[139] Herringa R. (2017). J. Trauma, PTSD, and the developing brain.. Curr. Psychiatry Rep..

[140] Paquola C., Bennett M.R., Lagopoulos J. (2016). Understanding heterogeneity in grey matter research of adults with childhood maltreatment-a meta-analysis and review.. Neurosci. Biobehav. Rev..

[141] Teicher M.H., Samson J.A., Anderson C.M., Ohashi K. (2016). The effects of childhood maltreatment on brain structure, function and connectivity.. Nat. Rev. Neurosci..

[142] Callaghan B.L., Tottenham N. (2016). The Stress Acceleration Hypothesis: Effects of early-life adversity on emotion circuits and behavior.. Curr. Opin. Behav. Sci..

[143] Gur R.E., Moore T.M., Rosen A.F.G., Barzilay R., Roalf D.R., Calkins M.E., Ruparel K., Scott J.C., Almasy L., Satterthwaite T.D., Shinohara R.T., Gur R.C. (2019). Burden of environmental adversity associated with psychopathology, maturation, and brain behavior parameters in youths.. JAMA Psychiatry.

[144] McLaughlin K.A., Weissman D., Bitrán D. (2019). Childhood adversity and neural development: A systematic review. Ann. Rev. Develop.. Psychol..

[145] Calem M., Bromis K., McGuire P., Morgan C., Kempton M.J. (2017). Meta-analysis of associations between childhood adversity and hippocampus and amygdala volume in non-clinical and general population samples.. Neuroimage Clin..

[146] Keding T.J., Heyn S.A., Russell J.D., Zhu X., Cisler J., McLaughlin K.A., Herringa R.J. (2021). Differential patterns of delayed emotion circuit maturation in abused girls with and without internalizing psychopathology.. Am. J. Psychiatry.

[147] Whittle S., Dennison M., Vijayakumar N., Simmons J.G., Yücel M., Lubman D.I., Pantelis C., Allen N.B. (2013). Childhood maltreatment and psychopathology affect brain development during adolescence.. J. Am. Acad. Child Adolesc. Psychiatry.

[148] Morey R.A., Haswell C.C., Hooper S.R., De Bellis M.D. (2016). Amygdala, hippocampus, and ventral medial prefrontal cortex volumes differ in maltreated youth with and without chronic posttraumatic stress disorder.. Neuropsychopharmacology.

[149] Keding T.J., Herringa R.J. (2015). Abnormal structure of fear circuitry in pediatric post-traumatic stress disorder.. Neuropsychopharmacology.

[150] Bremner J.D., Vythilingam M., Vermetten E., Southwick S.M., McGlashan T., Nazeer A., Khan S., Vaccarino L.V., Soufer R., Garg P.K., Ng C.K., Staib L.H., Duncan J.S., Charney D.S. (2003). MRI and PET study of deficits in hippocampal structure and function in women with childhood sexual abuse and posttraumatic stress disorder.. Am. J. Psychiatry.

[151] Whittle S., Lichter R., Dennison M., Vijayakumar N., Schwartz O., Byrne M.L., Simmons J.G., Yücel M., Pantelis C., McGorry P., Allen N.B. (2014). Structural brain development and depression onset during adolescence: a prospective longitudinal study.. Am. J. Psychiatry.

[152] Frodl T., Reinhold E., Koutsouleris N., Reiser M., Meisenzahl E.M. (2010). Interaction of childhood stress with hippocampus and prefrontal cortex volume reduction in major depression.. J. Psychiatr. Res..

[153] Gee D.G., Gabard-Durnam L.J., Flannery J., Goff B., Humphreys K.L., Telzer E.H., Hare T.A., Bookheimer S.Y., Tottenham N. (2013). Early developmental emergence of human amygdala-prefrontal connectivity after maternal deprivation.. Proc. Natl. Acad. Sci. USA.

[154] Rickard I.J., Frankenhuis W.E., Nettle D. (2014). Why are childhood family factors associated with timing of maturation? a role for internal prediction.. Perspect. Psychol. Sci..

[155] Herringa R.J., Burghy C.A., Stodola D.E., Fox M.E., Davidson R.J., Essex M.J. (2016). Enhanced prefrontal-amygdala connectivity following childhood adversity as a protective mechanism against internalizing in adolescence.. Biol. Psychiatry Cogn. Neurosci. Neuroimaging.

[156] Luby J.L., Tillman R., Barch D.M. (2019). Association of timing of adverse childhood experiences and caregiver support with regionally specific brain development in adolescents.. JAMA Netw. Open.

[157] Killion B.E., Weyandt L.L. (2020). Brain structure in childhood maltreatment-related PTSD across the lifespan: A systematic review.. Appl. Neuropsychol. Child.

[158] Opel N., Redlich R., Dohm K., Zaremba D., Goltermann J., Repple J., Kaehler C., Grotegerd D., Leehr E.J., Böhnlein J., Förster K., Meinert S., Enneking V., Sindermann L., Dzvonyar F., Emden D., Leenings R., Winter N., Hahn T., Kugel H., Heindel W., Buhlmann U., Baune B.T., Arolt V., Dannlowski U. (2019). Mediation of the influence of childhood maltreatment on depression relapse by cortical structure: A 2-year longitudinal observational study.. Lancet Psychiatry.

[159] Fujisawa T.X., Shimada K., Takiguchi S., Mizushima S., Kosaka H., Teicher M.H. (2018). Type and timing of childhood maltreatment and reduced visual cortex volume in children and adolescents with reactive attachment disorder.. Neuroimage Clin..

[160] Shimada K., Takiguchi S., Mizushima S., Fujisawa T.X., Saito D.N., Kosaka H., Okazawa H., Tomoda A. (2015). Reduced visual cortex grey matter volume in children and adolescents with reactive attachment disorder.. Neuroimage Clin..

[161] Madonna D., Delvecchio G., Soares J.C., Brambilla P. (2019). Structural and functional neuroimaging studies in generalized anxiety disorder: A systematic review.. Br. J. Psychiatry.

[162] Woon F.L., Sood S., Hedges D.W. (2010). Hippocampal volume deficits associated with exposure to psychological trauma and posttraumatic stress disorder in adults: A meta-analysis.. Prog. Neuropsychopharmacol. Biol. Psychiatry.

[163] Rauch S.L., Shin L.M., Phelps E.A. (2006). Neurocircuitry models of posttraumatic stress disorder and extinction: Human neuroimaging research—past, present, and future.. Biol. Psychiatry.

[164] Shin L.M., Rauch S.L., Pitman R.K. (2006). Amygdala, medial prefrontal cortex, and hippocampal function in PTSD.. In: Ann. N. Y. Acad. Sci..

[165] Tavanti M., Battaglini M., Borgogni F., Bossini L., Calossi S., Marino D., Vatti G., Pieraccini F., Federico A., Castrogiovanni P., De Stefano N. (2012). Evidence of diffuse damage in frontal and occipital cortex in the brain of patients with post-traumatic stress disorder.. Neurol. Sci..

[166] Bossini L., Santarnecchi E., Casolaro I., Koukouna D., Caterini C., Cecchini F., Fortini V., Vatti G., Marino D., Fernandez I., Rossi A., Fagiolini A. (2017). Morphovolumetric changes after EMDR treatment in drug-naïve PTSD patients.. Riv. Psichiatr..

[167] Nardo D., Högberg G., Looi J.C.L., Larsson S., Hällström T., Pagani M. (2010). Gray matter density in limbic and paralimbic cortices is associated with trauma load and EMDR outcome in PTSD patients.. J. Psychiatr. Res..

[168] Tan L., Zhang L., Qi R., Lu G., Li L., Liu J., Li W. (2013). Brain structure in post-traumatic stress disorder: A voxel-based morphometry analysis.. Neural Regen. Res..

[169] Zhang J., Tan Q., Yin H., Zhang X., Huan Y., Tang L., Wang H., Xu J., Li L. (2011). Decreased gray matter volume in the left hippocampus and bilateral calcarine cortex in coal mine flood disaster survivors with recent onset PTSD.. Psychiatry Res. Neuroimaging.

[170] Del Casale A., Ferracuti S., Barbetti A.S., Bargagna P., Zega P., Iannuccelli A., Caggese F., Zoppi T., De Luca G.P., Parmigiani G., Berardelli I., Pompili M. (2022). Grey matter volume reductions of the left hippocampus and amygdala in PTSD: A coordinate-based meta-analysis of magnetic resonance imaging studies.. Neuropsychobiology.

[171] Boccia M., D’Amico S., Bianchini F., Marano A., Giannini A.M., Piccardi L. (2016). Different neural modifications underpin PTSD after different traumatic events: An fMRI meta-analytic study.. Brain Imaging Behav..

[172] Baldaçara L., Borgio J.G.F., Araújo C., Nery-Fernandes F., Lacerda A.L.T., Moraes W.A.S., Montaño M.B.M.M., Rocha M., Quarantini L.C., Schoedl A., Pupo M., Mello M.F., Andreoli S.B., Miranda-Scippa A., Ramos L.R., Mari J.J., Bressan R.A., Jackowski A.P. (2012). Relationship between structural abnormalities in the cerebellum and dementia, posttraumatic stress disorder and bipolar disorder.. Dement. Neuropsychol..

[173] Tae W-S. (2015). Regional gray matter volume reduction associated with major depressive disorder: A voxel-based morphometry.. Investig. Magn. Reson. Imaging.

[174] Vythilingam M., Heim C., Newport J., Miller A.H., Anderson E., Bronen R., Brummer M., Staib L., Vermetten E., Charney D.S., Nemeroff C.B., Bremner J.D. (2002). Childhood trauma associated with smaller hippocampal volume in women with major depression.. Am. J. Psychiatry.

[175] Opel N., Redlich R., Zwanzger P., Grotegerd D., Arolt V., Heindel W., Konrad C., Kugel H., Dannlowski U. (2014). Hippocampal atrophy in major depression: A function of childhood maltreatment rather than diagnosis?. Neuropsychopharmacology.

[176] Soriano-Mas C., Hernández-Ribas R., Pujol J., Urretavizcaya M., Deus J., Harrison B.J., Ortiz H., López-Solà M., Menchón J.M., Cardoner N. (2011). Cross-sectional and longitudinal assessment of structural brain alterations in melancholic depression.. Biol. Psychiatry.

[177] Colle R., Dupong I., Colliot O., Deflesselle E., Hardy P., Falissard B., Ducreux D., Chupin M., Corruble E. (2018). Smaller hippocampal volumes predict lower antidepressant response/remission rates in depressed patients: A meta-analysis.. World J. Biol. Psychiatry.

[178] Gyger L., Ramponi C., Mall J.F., Swierkosz-Lenart K., Stoyanov D., Lutti A., von Gunten A., Kherif F., Draganski B. (2021). Temporal trajectory of brain tissue property changes induced by electroconvulsive therapy.. Neuroimage.

[179] Fonseka T.M., MacQueen G.M., Kennedy S.H. (2018). Neuroimaging biomarkers as predictors of treatment outcome in major depressive disorder.. J. Affect. Disord..

[180] Watanabe K., Kakeda S., Katsuki A., Ueda I., Ikenouchi A., Yoshimura R., Korogi Y. (2020). Whole-brain structural covariance network abnormality in first-episode and drug-naïve major depressive disorder.. Psychiatry Res. Neuroimaging.

[181] Shen Z., Cheng Y., Yang S., Dai N., Ye J., Liu X., Lu J., Li N., Liu F., Lu Y., Sun X., Xu X. (2016). Changes of grey matter volume in first-episode drug-naive adult major depressive disorder patients with different age-onset.. Neuroimage Clin..

[182] Espinoza Oyarce D.A., Shaw M.E., Alateeq K., Cherbuin N. (2020). Volumetric brain differences in clinical depression in association with anxiety: A systematic review with meta-analysis.. J. Psychiatry Neurosci..

[183] Hamilton J.P., Siemer M., Gotlib I.H. (2008). Amygdala volume in major depressive disorder: A meta-analysis of magnetic resonance imaging studies.. Mol. Psychiatry.

[184] Roddy D., Kelly J.R., Farrell C., Doolin K., Roman E., Nasa A., Frodl T., Harkin A., O’Mara S., O’Hanlon E., O’Keane V. (2021). Amygdala substructure volumes in Major Depressive Disorder.. Neuroimage Clin..

[185] Kim H., Han K.M., Choi K.W., Tae W.S., Kang W., Kang Y., Kim A., Ham B.J. (2021). Volumetric alterations in subregions of the amygdala in adults with major depressive disorder.. J. Affect. Disord..

[186] Wise T., Radua J., Via E., Cardoner N., Abe O., Adams T.M. (2015). Common and distinct patterns of grey-matter volume alteration in major depression and bipolar disorder: evidence from voxel-based meta-analysis.. Mol. Psychiatry.

[187] Jung J., Kang J., Won E., Nam K., Lee M.S., Tae W.S., Ham B.J. (2014). Impact of lingual gyrus volume on antidepressant response and neurocognitive functions in Major Depressive Disorder: A voxel-based morphometry study.. J. Affect. Disord..

[188] Lai C.H., Wu Y.T. (2014). Frontal-insula gray matter deficits in first-episode medication-naïve patients with major depressive disorder.. J. Affect. Disord..

[189] Abe O., Yamasue H., Kasai K., Yamada H., Aoki S., Inoue H., Takei K., Suga M., Matsuo K., Kato T., Masutani Y., Ohtomo K. (2010). Voxel-based analyses of gray/white matter volume and diffusion tensor data in major depression.. Psychiatry Res. Neuroimaging.

[190] Vasic N., Walter H., Höse A., Wolf R.C. (2008). Gray matter reduction associated with psychopathology and cognitive dysfunction in unipolar depression: A voxel-based morphometry study.. J. Affect. Disord..

[191] Leung K.K., Lee T.M.C., Wong M.M.C., Li L.S.W., Yip P.S.F., Khong P.L. (2009). Neural correlates of attention biases of people with major depressive disorder: A voxel-based morphometric study.. Psychol. Med..

[192] Serra-Blasco M., Portella M.J., Gómez-Ansón B., de Diego-Adeliño J., Vives-Gilabert Y., Puigdemont D., Granell E., Santos A., Álvarez E., Pérez V. (2013). Effects of illness duration and treatment resistance on grey matter abnormalities in majordepression.. Br. J. Psychiatry.

[193] Chen C., Liu Z., Xi C., Tan W., Fan Z., Cheng Y., Yang J., Palaniyappan L., Yang J. (2022). Multimetric structural covariance in first-episode major depressive disorder: A graph theoretical analysis.. J. Psychiatry Neurosci..

[194] Kandilarova S., Stoyanov D., Sirakov N., Maes M., Specht K. (2019). Reduced grey matter volume in frontal and temporal areas in depression: Contributions from voxel-based morphometry study.. Acta Neuropsychiatr..

[195] Schmaal L., Hibar D.P., Sämann P.G., Hall G.B., Baune B.T., Jahanshad N., Cheung J.W., van Erp T.G.M., Bos D., Ikram M.A., Vernooij M.W., Niessen W.J., Tiemeier H., Hofman A., Wittfeld K., Grabe H.J., Janowitz D., Bülow R., Selonke M., Völzke H., Grotegerd D., Dannlowski U., Arolt V., Opel N., Heindel W., Kugel H., Hoehn D., Czisch M., Couvy-Duchesne B., Rentería M.E., Strike L.T., Wright M.J., Mills N.T., de Zubicaray G.I., McMahon K.L., Medland S.E., Martin N.G., Gillespie N.A., Goya-Maldonado R., Gruber O., Krämer B., Hatton S.N., Lagopoulos J., Hickie I.B., Frodl T., Carballedo A., Frey E.M., van Velzen L.S., Penninx B.W.J.H., van Tol M-J., van der Wee N.J., Davey C.G., Harrison B.J., Mwangi B., Cao B., Soares J.C., Veer I.M., Walter H., Schoepf D., Zurowski B., Konrad C., Schramm E., Normann C., Schnell K., Sacchet M.D., Gotlib I.H., MacQueen G.M., Godlewska B.R., Nickson T., McIntosh A.M., Papmeyer M., Whalley H.C., Hall J., Sussmann J.E., Li M., Walter M., Aftanas L., Brack I., Bokhan N.A., Thompson P.M., Veltman D.J. (2017). Cortical abnormalities in adults and adolescents with major depression based on brain scans from 20 cohorts worldwide in the ENIGMA Major Depressive Disorder Working Group.. Mol. Psychiatry.

[196] Grieve S.M., Korgaonkar M.S., Koslow S.H., Gordon E., Williams L.M. (2013). Widespread reductions in gray matter volume in depression.. Neuroimage Clin..

[197] Salvadore G., Nugent A.C., Lemaitre H., Luckenbaugh D.A., Tinsley R., Cannon D.M., Neumeister A., Zarate C.A., Drevets W.C. (2011). Prefrontal cortical abnormalities in currently depressed versus currently remitted patients with major depressive disorder.. Neuroimage.

[198] Machino A., Kunisato Y., Matsumoto T., Yoshimura S., Ueda K., Yamawaki Y., Okada G., Okamoto Y., Yamawaki S. (2014). Possible involvement of rumination in gray matter abnormalities in persistent symptoms of major depression: An exploratory magnetic resonance imaging voxel-based morphometry study.. J. Affect. Disord..

[199] Serra-Blasco M., de Diego-Adeliño J., Vives-Gilabert Y., Trujols J., Puigdemont D., Carceller-Sindreu M., Pérez V., Álvarez E., Portella M.J. (2016). Naturalistic course of major depressive disorder predicted by clinical and structural neuroimaging data: A 5-Year Follow-Up.. Depress. Anxiety.

[200] Costafreda S.G., Chu C., Ashburner J., Fu C.H.Y. (2009). Prognostic and diagnostic potential of the structural neuroanatomy of depression.. PLoS One.

[201] Caetano S.C., Kaur S., Brambilla P., Nicoletti M., Hatch J.P., Sassi R.B., Mallinger A.G., Keshavan M.S., Kupfer D.J., Frank E., Soares J.C. (2006). Smaller cingulate volumes in unipolar depressed patients.. Biol. Psychiatry.

[202] Leech R., Sharp D.J. (2014). The role of the posterior cingulate cortex in cognition and disease.. Brain.

[203] Greicius M.D., Krasnow B., Reiss A.L., Menon V. (2003). Functional connectivity in the resting brain: A network analysis of the default mode hypothesis.. Proc. Natl. Acad. Sci. USA.

[204] Rolls E.T. (2019). The cingulate cortex and limbic systems for emotion, action, and memory.. Brain Structure and Function.

[205] Yeh P.H., Zhu H., Nicoletti M.A., Hatch J.P., Brambilla P., Soares J.C. (2010). Structural equation modeling and principal component analysis of gray matter volumes in major depressive and bipolar disorders: Differences in latent volumetric structure.. Psychiatry Res. Neuroimaging.

[206] Serra-Blasco M., Lam R.W., Harmer C.J., Baune B.T. (2019). Clinical and functional characteristics of cognitive dysfunction in major depressive disorder.. Cognitive Dimensions of Major Depressive Disorder..

[207] López-Solà C., Subirà M., Serra-Blasco M., Vicent-Gil M., Navarra-Ventura G., Aguilar E., Acebillo S., Palao D.J., Cardoner N. (2020). Is cognitive dysfunction involved in difficult-to-treat depression? Characterizing resistance from a cognitive perspective.. Eur. Psychiatry.

[208] Serra-Blasco M., de Vita S., Rodríguez M.R., de Diego-Adeliño J., Puigdemont D., Martín-Blanco A., Pérez-Egea R., Molet J., Álvarez E., Pérez V., Portella M.J. (2015). Cognitive functioning after deep brain stimulation in subcallosal cingulate gyrus for treatment-resistant depression: An exploratory study.. Psychiatry Res..

[209] Cui L., Wang F., Yin Z., Chang M., Song Y., Wei Y., Lv J., Zhang Y., Tang Y., Gong X., Xu K. (2020). Effects of the LHPP gene polymorphism on the functional and structural changes of gray matter in major depressive disorder.. Quant. Imaging Med. Surg..

[210] Gogolla N. (2017). The insular cortex.. Current Biology.

[211] Zhao Y., Chen L., Zhang W., Xiao Y., Shah C., Zhu H., Yuan M., Sun H., Yue Q., Jia Z., Zhang W., Kuang W., Gong Q., Lui S. (2017). Gray matter abnormalities in non-comorbid medication-naive patients with major depressive disorder or social anxiety disorder.. EBioMedicine.

[212] Zhang Y., Yang Y., Zhu L., Zhu Q., Jia Y., Zhang L. (2021). Volumetric deficit within the fronto-limbic-striatal circuit in first-episode drug naïve patients with major depression disorder.. Front. Psychiatry.

[213] Ge R., Hassel S., Arnott S.R., Davis A.D., Harris J.K., Zamyadi M., Milev R., Frey B.N., Strother S.C., Müller D.J., Rotzinger S., MacQueen G.M., Kennedy S.H., Lam R.W., Vila-Rodriguez F. (2021). Structural covariance pattern abnormalities of insula in major depressive disorder: A CAN-BIND study report.. Prog. Neuropsychopharmacol. Biol. Psychiatry.

[214] Yang J., Yin Y., Svob C., Long J., He X., Zhang Y., Xu Z., Li L., Liu J., Dong J., Zhang Z., Wang Z., Yuan Y. (2017). Amygdala atrophy and its functional disconnection with the cortico-striatal-pallidal-thalamic circuit in major depressive disorder in females.. PLoS One.

[215] Wise T., Radua J., Nortje G., Cleare A.J., Young A.H., Arnone D. (2016). Voxel-based meta-Analytical evidence of structural disconnectivity in major depression and bipolar disorder.. Biol. Psychiatry.

[216] Peng J., Liu J., Nie B., Li Y., Shan B., Wang G., Li K. (2011). Cerebral and cerebellar gray matter reduction in first-episode patients with major depressive disorder: A voxel-based morphometry study.. Eur. J. Radiol..

[217] Lee H.Y., Tae W.S., Yoon H.K., Lee B.T., Paik J.W., Son K.R., Oh Y.W., Lee M.S., Ham B.J. (2011). Demonstration of decreased gray matter concentration in the midbrain encompassing the dorsal raphe nucleus and the limbic subcortical regions in major depressive disorder: An optimized voxel-based morphometry study.. J. Affect. Disord..

[218] Han S., Zheng R., Li S., Liu L., Wang C., Jiang Y., Wen M., Zhou B., Wei Y., Pang J., Li H., Zhang Y., Chen Y., Cheng J. (2023). Progressive brain structural abnormality in depression assessed with MR imaging by using causal network analysis.. Psychol. Med..

[219] Depping M.S., Schmitgen M.M., Kubera K.M., Wolf R.C. (2018). Cerebellar Contributions to Major Depression; Frontiers in Psychiatry.. Frontiers Media S.A..

[220] Friedman M.J., Resick P.A., Bryant R.A., Strain J., Horowitz M., Spiegel D. (2011). Classification of trauma and stressor-related disorders in DSM-5.. Depress. Anxiety.

[221] Bandelow B., Baldwin D., Abelli M., Altamura C., Dell’Osso B., Domschke K., Fineberg N.A., Grünblatt E., Jarema M., Maron E., Nutt D., Pini S., Vaghi M.M., Wichniak A., Zai G., Riederer P. (2016). Biological markers for anxiety disorders, OCD and PTSD-a consensus statement. Part I: Neuroimaging and genetics.. World J. Biol. Psychiatry.

[222] Norton P.J., Paulus D.J. (2017). Transdiagnostic models of anxiety disorder: Theoretical and empirical underpinnings.. Clin. Psychol. Rev..

[223] Hur J., Smith J.F., DeYoung K.A., Anderson A.S., Kuang J., Kim H.C., Tillman R.M., Kuhn M., Fox A.S., Shackman A.J. (2020). Anxiety and the neurobiology of temporally uncertain threat anticipation.. J. Neurosci..

[224] Chen Y.H., Wu J.L., Hu N.Y., Zhuang J.P., Li W.P., Zhang S.R., Li X.W., Yang J.M., Gao T.M. (2021). Distinct projections from the infralimbic cortex exert opposing effects in modulating anxiety and fear.. J. Clin. Invest..

[225] Huggins A.A., Weis C.N., Parisi E.A., Bennett K.P., Miskovic V., Larson C.L. (2021). Neural substrates of human fear generalization: A 7T-fMRI investigation.. Neuroimage.

[226] Sangha S., Diehl M.M., Bergstrom H.C., Drew M.R. (2020). Know safety, no fear.. Neurosci. Biobehav. Rev..

[227] Bian X.L., Qin C., Cai C.Y., Zhou Y., Tao Y., Lin Y.H., Wu H.Y., Chang L., Luo C.X., Zhu D.Y. (2019). Anterior cingulate cortex to ventral hippocampus circuit mediates contextual fear generalization.. J. Neurosci..

[228] Mah L., Szabuniewicz C., Fiocco A.J. (2016). Can anxiety damage the brain?. Curr. Opin. Psychiatry.

[229] Kolesar T.A., Bilevicius E., Wilson A.D., Kornelsen J. (2019). Systematic review and meta-analyses of neural structural and functional differences in generalized anxiety disorder and healthy controls using magnetic resonance imaging.. Neuroimage Clin..

[230] Moon C.M., Jeong G.W. (2017). Abnormalities in gray and white matter volumes associated with explicit memory dysfunction in patients with generalized anxiety disorder.. Acta Radiol..

[231] Chen Y., Cui Q., Fan Y.S., Guo X., Tang Q., Sheng W., Lei T., Li D., Lu F., He Z., Yang Y., Hu S., Deng J., Chen H. (2020). Progressive brain structural alterations assessed via causal analysis in patients with generalized anxiety disorder.. Neuropsychopharmacology.

[232] Takaishi M., Asami T., Yoshida H., Nakamura R., Yoshimi A., Hirayasu Y. (2021). Smaller volume of right hippocampal CA2/3 in patients with panic disorder.. Brain Imaging Behav..

[233] Sheng L.Q., Ma H.R., Yao L.Z., Dai Z.Y. (2020). Consistent brain grey matter volume alterations in adult patients with panic disorder and social anxiety disorder revisited.. J. Affect. Disord..

[234] Wang X., Cheng B., Wang S., Lu F., Luo Y., Long X., Kong D. (2021). Distinct grey matter volume alterations in adult patients with panic disorder and social anxiety disorder: A systematic review and voxel-based morphometry meta-analysis.. J. Affect. Disord..

[235] Rauch S.L., Shin L.M., Wright C. (2003). Neuroimaging studies of amygdala function in anxiety disorders.. Ann. N. Y. Acad. Sci..

[236] Etkin A., Wager T.D. (2007). Functional neuroimaging of anxiety: A meta-analysis of emotional processing in PTSD, social anxiety disorder, and specific phobia.. Am. J. Psychiatry.

[237] Davis M. (1992). The role of the amygdala in fear and anxiety.. Annu. Rev. Neurosci..

[238] Britton J.C., Rauch S.L. (2018). Neuroanatomy and Neuroimaging of Anxiety Disorders..

[239] Massana G., Serra-Grabulosa J.M., Salgado-Pineda P., Gastó C., Junqué C., Massana J., Mercader J.M., Gómez B., Tobeña A., Salamero M. (2003). Amygdalar atrophy in panic disorder patients detected by volumetric magnetic resonance imaging.. Neuroimage.

[240] Asami T., Yamasue H., Hayano F., Nakamura M., Uehara K., Otsuka T., Roppongi T., Nihashi N., Inoue T., Hirayasu Y. (2009). Sexually dimorphic gray matter volume reduction in patients with panic disorder.. Psychiatry Res. Neuroimaging.

[241] Asami T., Nakamura R., Takaishi M., Yoshida H., Yoshimi A., Whitford T.J. (2018). Smaller volumes in the lateral and basal nuclei of the amygdala in patients with panic disorder.. PLoS One.

[242] Hayano F., Nakamura M., Asami T., Uehara K., Yoshida T., Roppongi T., Otsuka T., Inoue T., Hirayasu Y. (2009). Smaller amygdala is associated with anxiety in patients with panic disorder.. Psychiatry Clin. Neurosci..

[243] Kunas S.L., Hilbert K., Yang Y., Richter J., Hamm A., Wittmann A., Ströhle A., Pfleiderer B., Herrmann M.J., Lang T., Lotze M., Deckert J., Arolt V., Wittchen H.U., Straube B., Kircher T., Gerlach A.L., Lueken U. (2020). The modulating impact of cigarette smoking on brain structure in panic disorder: A voxel-based morphometry study.. Soc. Cogn. Affect. Neurosci..

[244] Hilbert K., Lueken U., Beesdo-Baum K. (2014). Neural structures, functioning and connectivity in Generalized Anxiety Disorder and interaction with neuroendocrine systems: A systematic review.. J. Affect. Disord..

[245] Makovac E., Meeten F., Watson D.R., Garfinkel S.N., Critchley H.D., Ottaviani C. (2016). Neurostructural abnormalities associated with axes of emotion dysregulation in generalized anxiety.. Neuroimage Clin..

[246] Ma Z., Wang C., Hines C.S., Lu X., Wu Y., Xu H., Li J., Wang Q., Pang M., Zhong Y., Zhang N. (2019). Frontoparietal network abnormalities of gray matter volume and functional connectivity in patients with generalized anxiety disorder.. Psychiatry Res. Neuroimaging.

[247] Bas-Hoogendam J.M., van Steenbergen H., Tissier R.L.M., Houwing-Duistermaat J.J., Westenberg P.M., van der Wee N.J.A. (2018). Subcortical brain volumes, cortical thickness and cortical surface area in families genetically enriched for social anxiety disorder - A multiplex multigenerational neuroimaging study.. EBioMedicine.

[248] Fisler M.S., Federspiel A., Horn H., Dierks T., Schmitt W., Wiest R., de Quervain D.J.F., Soravia L.M. (2013). Spider phobia is associated with decreased left amygdala volume: A cross-sectional study.. BMC Psychiatry.

[249] Hilbert K., Evens R., Isabel M.N., Wittchen H.U., Lueken U. (2015). Neurostructural correlates of two subtypes of specific phobia: A voxel-based morphometry study.. Psychiatry Res. Neuroimaging.

[250] Linares I.M.P., Jackowski A.P., Trzesniak C.M.F., Arrais K.C., Chagas M.H.N., Sato J.R., Santos A.C., Hallak J.E.C., Zuardi A.W., Nardi A.E., Coimbra N.C., Crippa J.A.S. (2014). Cortical thinning of the right anterior cingulate cortex in spider phobia: A magnetic resonance imaging and spectroscopy study.. Brain Res..

[251] Rauch S.L., Wright C.I., Martis B., Busa E., McMullin K.G., Shin L.M., Dale A.M., Fischl B. (2004). A magnetic resonance imaging study of cortical thickness in animal phobia.. Biol. Psychiatry.

[252] Fareri D.S., Tottenham N. (2016). Effects of early life stress on amygdala and striatal development.. Dev. Cogn. Neurosci..

[253] Brandl F., Weise B., Mulej Bratec S., Jassim N., Hoffmann A.D., Bertram T., Ploner M., Sorg C. (2022). Common and specific large-scale brain changes in major depressive disorder, anxiety disorders, and chronic pain: A transdiagnostic multimodal meta-analysis of structural and functional MRI studies.. Neuropsychopharmacology.

[254] Adhikari A., Lerner T.N., Finkelstein J., Pak S., Jennings J.H., Davidson T.J., Ferenczi E., Gunaydin L.A., Mirzabekov J.J., Ye L., Kim S.Y., Lei A., Deisseroth K. (2015). Basomedial amygdala mediates top-down control of anxiety and fear.. Nature.

[255] Shiba Y., Santangelo A.M., Roberts A.C., Adhikari A., Bissonette G.B. (2016). Rudebeck, pH beyond the medial regions of prefrontal cortex in the regulation of fear and anxiety.. Front. Syst. Neurosci..

[256] Duval E.R., Javanbakht A., Liberzon I. (2015). Neural circuits in anxiety and stress disorders: A focused review.. Ther. Clin. Risk Manag..

[257] Cho J.H., Deisseroth K., Bolshakov V.Y. (2013). Synaptic encoding of fear extinction in mPFC-amygdala circuits.. Neuron.

[258] Schiller D., Delgado M.R. (2010). Overlapping neural systems mediating extinction, reversal and regulation of fear.. Trends Cogn. Sci..

[259] Wang H.Y., Zhang X.X., Si C.P., Xu Y., Liu Q., Bian H.T., Zhang B.W., Li X.L., Yan Z.R. (2018). Prefrontoparietal dysfunction during emotion regulation in anxiety disorder: A meta-analysis of functional magnetic resonance imaging studies.. Neuropsychiatr. Dis. Treat..

[260] Killgore W.D.S., Britton J.C., Schwab Z.J., Price L.M., Weiner M.R., Gold A.L., Rosso I.M., Simon N.M., Pollack M.H., Rauch S.L. (2014). Cortico-limbic responses to masked affective faces across ptsd, panic disorder, and specific phobia.. Depress. Anxiety.

[261] Liu W.Z., Zhang W.H., Zheng Z.H., Zou J.X., Liu X.X., Huang S.H., You W.J., He Y., Zhang J.Y., Wang X.D., Pan B.X. (2020). Identification of a prefrontal cortex-to-amygdala pathway for chronic stress-induced anxiety.. Nat. Commun..

[262] Shang J., Fu Y., Ren Z., Zhang T., Du M., Gong Q., Lui S., Zhang W. (2014). The common traits of the ACC and PFC in anxiety disorders in the DSM-5: Meta-analysis of voxel-based morphometry studies.. PLoS One.

[263] Li H., Zhang B., Hu Q., Zhang L., Jin Y., Wang J., Cui H., Pang J., Li C. (2020). Altered heartbeat perception sensitivity associated with brain structural alterations in generalised anxiety disorder.. Gen. Psychiatr..

[264] Andreescu C., Tudorascu D., Sheu L.K., Rangarajan A., Butters M.A., Walker S., Berta R., Desmidt T., Aizenstein H. (2017). Brain structural changes in late-life generalized anxiety disorder.. Psychiatry Res. Neuroimaging.

[265] Harrewijn A., Cardinale E.M., Groenewold N.A., Bas-Hoogendam J.M., Aghajani M., Hilbert K., Cardoner N., Porta-Casteràs D., Gosnell S., Salas R., Jackowski A.P., Pan P.M., Salum G.A., Blair K.S., Blair J.R., Hammoud M.Z., Milad M.R., Burkhouse K.L., Phan K.L., Schroeder H.K., Strawn J.R., Beesdo-Baum K., Jahanshad N., Thomopoulos S.I., Buckner R., Nielsen J.A., Smoller J.W., Soares J.C., Mwangi B., Wu M.J., Zunta-Soares G.B., Assaf M., Diefenbach G.J., Brambilla P., Maggioni E., Hofmann D., Straube T., Andreescu C., Berta R., Tamburo E., Price R.B., Manfro G.G., Agosta F., Canu E., Cividini C., Filippi M. (2021). Kostić M.; Munjiza Jovanovic, A.; Alberton, B.A.V.; Benson, B.; Freitag, G.F.; Filippi, C.A.; Gold, A.L.; Leibenluft, E.; Ringlein, G.V.; Werwath, K.E.; Zwiebel, H.; Zugman, A.; Grabe, H.J.; Van der Auwera, S.; Wittfeld, K.; Völzke, H.; Bülow, R.; Balderston, N.L.; Ernst, M.; Grillon, C.; Mujica-Parodi, L.R.; van Nieuwenhuizen, H.; Critchley, H.D.; Makovac, E.; Mancini, M.; Meeten, F.; Ottaviani, C.; Ball, T.M.; Fonzo, G.A.; Paulus, M.P.; Stein, M.B.; Gur, R.E.; Gur, R.C.; Kaczkurkin, A.N.; Larsen, B.; Satterthwaite, T.D.; Harper, J.; Myers, M.; Perino, M.T.; Sylvester, C.M.; Yu, Q.; Lueken, U.; Veltman, D.J.; Thompson, P.M.; Stein, D.J.; Van der Wee, N.J.A.; Winkler, A.M.; Pine, D.S. Cortical and subcortical brain structure in generalized anxiety disorder: findings from 28 research sites in the ENIGMA-Anxiety Working Group.. Transl. Psychiatry.

[266] Na K.S., Ham B.J., Lee M.S., Kim L., Kim Y.K., Lee H.J., Yoon H.K. (2013). Decreased gray matter volume of the medial orbitofrontal cortex in panic disorder with agoraphobia: A preliminary study.. Prog. Neuropsychopharmacol. Biol. Psychiatry.

[267] Uchida R.R., Del-Ben C.M., Busatto G.F., Duran F.L.S., Guimarães F.S., Crippa J.A.S., Araújo D., Santos A.C., Graeff F.G. (2008). Regional gray matter abnormalities in panic disorder: A voxel-based morphometry study.. Psychiatry Res. Neuroimaging.

[268] Ni M.F., Wang X.M., Wang H.Y., Chang Y., Huang X.F., Zhang B.W. (2020). Regional cortical thinning and cerebral hypoperfusion in patients with panic disorder.. J. Affect. Disord..

[269] Liao W., Xu Q., Mantini D., Ding J., Machado-de-Sousa J.P., Hallak J.E.C., Trzesniak C., Qiu C., Zeng L., Zhang W., Crippa J.A.S., Gong Q., Chen H. (2011). Altered gray matter morphometry and resting-state functional and structural connectivity in social anxiety disorder.. Brain Res..

[270] Zhang X., Luo Q., Wang S., Qiu L., Pan N., Kuang W., Lui S., Huang X., Yang X., Kemp G.J., Gong Q. (2020). Dissociations in cortical thickness and surface area in non-comorbid never-treated patients with social anxiety disorder.. EBioMedicine.

[271] Bas-Hoogendam J.M., van Steenbergen H., Nienke Pannekoek J., Fouche J.P., Lochner C., Hattingh C.J., Cremers H.R., Furmark T., Månsson K.N.T., Frick A., Engman J., Boraxbekk C.J., Carlbring P., Andersson G., Fredrikson M., Straube T., Peterburs J., Klumpp H., Phan K.L., Roelofs K., Veltman D.J., van Tol M.J., Stein D.J., van der Wee N.J.A. (2017). Voxel-based morphometry multi-center mega-analysis of brain structure in social anxiety disorder.. Neuroimage Clin..

[272] Lai C.H., Wu Y.T. (2015). The gray matter alterations in major depressive disorder and panic disorder: Putative differences in the pathogenesis.. J. Affect. Disord..

[273] Atmaca M., Koc M., Mermi O., Korkmaz S., Aslan S., Yildirim H. (2021). Insula volumes are altered in patients with social anxiety disorder.. Behav. Brain Res..

[274] Kawaguchi A., Nemoto K., Nakaaki S., Kawaguchi T., Kan H., Arai N., Shiraishi N., Hashimoto N., Akechi T. (2016). Insular volume reduction in patients with social anxiety disorder.. Front. Psychiatry.

[275] Syal S., Hattingh C.J., Fouché J.P., Spottiswoode B., Carey P.D., Lochner C., Stein D.J. (2012). Grey matter abnormalities in social anxiety disorder: A pilot study.. Metab. Brain Dis..

[276] Marchand W.R. (2010). Cortico-basal ganglia circuitry: A review of key research and implications for functional connectivity studies of mood and anxiety disorders.. Brain Struct. Funct..

[277] Zhang X., Suo X., Yang X., Lai H., Pan N., He M., Li Q., Kuang W., Wang S., Gong Q. (2022). Structural and functional deficits and couplings in the cortico-striato-thalamo-cerebellar circuitry in social anxiety disorder.. Transl. Psychiatry.

[278] Yoo H.K., Kim M.J., Kim S.J., Sung Y.H., Sim M.E., Lee Y.S., Song S.Y., Kee B.S., Lyoo I.K. (2005). Putaminal gray matter volume decrease in panic disorder: An optimized voxel-based morphometry study.. Eur. J. Neurosci..

[279] Yoshida H., Asami T., Takaishi M., Nakamura R., Yoshimi A., Whitford T.J., Hirayasu Y. (2020). Structural abnormalities in nucleus accumbens in patients with panic disorder.. J. Affect. Disord..

[280] Wang X., Cheng B., Luo Q., Qiu L., Wang S. (2018). Gray matter structural alterations in social anxiety disorder: A voxel-based meta-analysis.. Front. Psychiatry.

[281] Asami T., Yoshida H., Takaishi M., Nakamura R., Yoshimi A., Whitford T.J., Hirayasu Y. (2018). Thalamic shape and volume abnormalities in female patients with panic disorder.. PLoS One.

[282] Talati A., Pantazatos S.P., Schneier F.R., Weissman M.M., Hirsch J. (2013). Gray matter abnormalities in social anxiety disorder: Primary, replication, and specificity studies.. Biol. Psychiatry.

[283] Tükel R. (2015). Aydın, K.; Yüksel, Ç.; Ertekin, E.; Koyuncu, A.; Taş C. Gray matter abnormalities in patients with social anxiety disorder: A voxel-based morphometry study.. Psychiatry Res. Neuroimaging.

[284] Pujol J., Harrison B.J., Ortiz H., Deus J., Soriano-Mas C., López-Solà M., Yücel M., Perich X., Cardoner N. (2009). Influence of the fusiform gyrus on amygdala response to emotional faces in the non-clinical range of social anxiety.. Psychol. Med..

[285] Cuthbert B.N., Insel T.R. (2013). Toward the future of psychiatric diagnosis: The seven pillars of RDoC.. BMC Med..

[286] Howard D.M., Adams M.J., Clarke T.K., Hafferty J.D., Gibson J., Shirali M., Coleman J.R.I., Hagenaars S.P., Ward J., Wigmore E.M., Alloza C., Shen X., Barbu M.C., Xu E.Y., Whalley H.C., Marioni R.E., Porteous D.J., Davies G., Deary I.J., Hemani G., Berger K., Teismann H., Rawal R., Arolt V., Baune B.T., Dannlowski U., Domschke K., Tian C., Hinds D.A., Trzaskowski M., Byrne E.M., Ripke S., Smith D.J., Sullivan P.F., Wray N.R., Breen G., Lewis C.M., McIntosh A.M. (2019). Genome-wide meta-analysis of depression identifies 102 independent variants and highlights the importance of the prefrontal brain regions.. Nat. Neurosci..

[287] Schiele M.A., Gottschalk M.G., Domschke K. (2020). The applied implications of epigenetics in anxiety, affective and stress-related disorders - A review and synthesis on psychosocial stress, psychotherapy and prevention.. Clin. Psychol. Rev..

[288] Pfeiffer J.R., Mutesa L., Uddin M. (2018). Traumatic stress epigenetics.. Curr. Behav. Neurosci. Rep..

[289] Jawaid A., Roszkowski M., Mansuy I.M. (2018). Transgenerational epigenetics of traumatic stress.. Prog. Mol. Biol. Transl. Sci..

[290] Dupont C., Armant D., Brenner C. (2009). Epigenetics: Definition, mechanisms and clinical perspective.. Semin. Reprod. Med..

[291] Zugman A., Harrewijn A., Cardinale E.M., Zwiebel H., Freitag G.F., Werwath K.E. (2020). Mega-analysis methods in enigma: The experience of the generalized anxiety disorder working group.. Hum. Brain Mapp..

[292] Lijffijt M., Green C.E., Balderston N., Iqbal T., Atkinson M. (2019). Vo-Le, Brittany, B.; Vo-Le, Bylinda, B.; O’Brien, B.; Grillon, C.; Swann, A.C.; Mathew, S.J. A proof-of-mechanism study to test effects of the NMDA receptor antagonist lanicemine on behavioral sensitization in individuals with symptoms of PTSD.. Front. Psychiatry.

[293] Newport D.J., Carpenter L.L., McDonald W.M., Potash J.B., Tohen M., Nemeroff C.B. (2015). Ketamine and other NMDA antagonists: Early clinical trials and possible mechanisms in depression.. Am. J. Psychiatry.

[294] Ates-Alagoz Z., Adejare A. (2013). NMDA receptor antagonists for treatment of depression.. Pharmaceuticals.

[295] Lorigooini Z., Nasiri B.S., Balali-Dehkordi S., Ebrahimi L., Bijad E., Rahimi-Madiseh M., Amini-Khoei H. (2020). Possible involvement of NMDA receptor in the anxiolytic-like effect of caffeic acid in mice model of maternal separation stress.. Heliyon.

